# Guidelines for Regulated Cell Death Assays: A Systematic Summary, A Categorical Comparison, A Prospective

**DOI:** 10.3389/fcell.2021.634690

**Published:** 2021-03-04

**Authors:** Xi-min Hu, Zhi-xin Li, Rui-han Lin, Jia-qi Shan, Qing-wei Yu, Rui-xuan Wang, Lv-shuang Liao, Wei-tao Yan, Zhen Wang, Lei Shang, Yanxia Huang, Qi Zhang, Kun Xiong

**Affiliations:** ^1^Department of Anatomy and Neurobiology, School of Basic Medical Sciences, Central South University, Changsha, China; ^2^Wuxi School of Medicine, Jiangnan University, Wuxi, China; ^3^Jiangxi Research Institute of Ophthalmology and Visual Sciences, Affiliated Eye Hospital of Nanchang University, Nanchang, China; ^4^Hunan Key Laboratory of Ophthalmology, Changsha, China

**Keywords:** regulated cell death, detecting methods, guidelines, biomarkers, clinical application

## Abstract

Over the past few years, the field of regulated cell death continues to expand and novel mechanisms that orchestrate multiple regulated cell death pathways are being unveiled. Meanwhile, researchers are focused on targeting these regulated pathways which are closely associated with various diseases for diagnosis, treatment, and prognosis. However, the complexity of the mechanisms and the difficulties of distinguishing among various regulated types of cell death make it harder to carry out the work and delay its progression. Here, we provide a systematic guideline for the fundamental detection and distinction of the major regulated cell death pathways following morphological, biochemical, and functional perspectives. Moreover, a comprehensive evaluation of different assay methods is critically reviewed, helping researchers to make a reliable selection from among the cell death assays. Also, we highlight the recent events that have demonstrated some novel regulated cell death processes, including newly reported biomarkers (e.g., non-coding RNA, exosomes, and proteins) and detection techniques.

## Introduction

Cell death is generally classified into two types: accidental cell death (ACD) and regulated cell death (RCD). RCD is regarded as reversible and can be blocked by small inhibitors (Wang S. et al., 2017; Cheng et al., 2018; Kumar and Sandhir, 2018; Wang et al., 2019c, d). Various programs of RCD have been described and their research continues to progress, including apoptosis (extrinsic and intrinsic), regulated necrosis (namely necroptosis), autophagy-dependent cell death (e.g., autosis), pyroptosis, ferroptosis, NETosis, parthanatos, entotic cell death, anoikis, lysosome-dependent cell death, and mitotic death (Kroemer et al., 2005; Galluzzi et al., 2018). Both in technical research and mechanism mining, the research into these types of RCDs has made great progress, and we have selected in-depth research and rapid recent development of the knowledge of RCDs as an illustration, including apoptosis, necroptosis, autophagy, pyroptosis, ferroptosis, and NETosis.

With the development of RCD studies, the detection methods have been improved simultaneously and diversified for accurate identification and systematic analysis. The history of RCDs began in 1842 when dying cells were noticed by Karl Vogt in toads (Tang et al., 2019). In Lockshin and Williams (1964) reported an orderly and predictable pattern of birth and death at the cellular level, called metamorphosis, a phenomenon with pycnotic nuclei, shrunken and degenerated mitochondria, and conspicuous lysosome-like bodies observed *via* light and electron microscopy (EM) (Lockshin and Williams, 1964, 1965). Apoptosis was termed “shrinkage necrosis” in Kerr (1971); Kerr et al. (1972) distinguished two types of cell death (apoptosis and necrosis) in human pathology samples, focusing on cell morphology, and described necrotic cells as swollen cells with swollen organelles.

The first description of pyroptosis was reported in Zychlinsky et al. (1992), but the term “pyroptosis” was first coined in Cookson and Brennan (2001) after an observation of bacteria-infected macrophages going through a rapidly caspase 1-dependent lytic cell death pathway.

**FIGURE 1:**
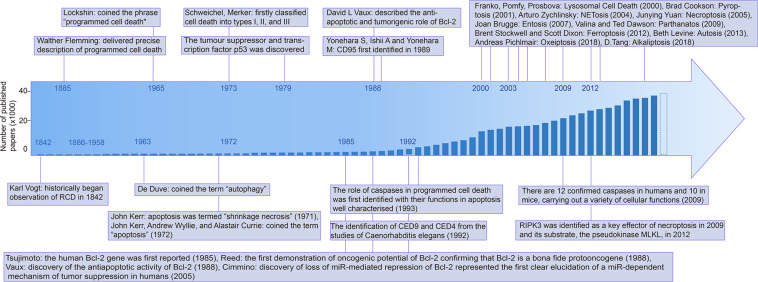
Timeline of the mile stone of cell death research. Abbreviations: Bcl-2: B-cell leukemia/lymphoma-2, CED-9: Cell death abnormality gene 9, RIPK3: Receptor-interacting serine/threonine-protein kinase 3, MLKL: Mixed lineage kinase domain like pseudokinase, PCD: Programmed cell death, CD95: cluster of differentiation 95.

In the early 21st century, necrosis was previously considered to be uncontrollable, but it was recently revised as a partly regulated mechanism, namely necroptosis, involving mitochondrial permeability transition through morphological and biochemical detection (Vercammen et al., 1998; Holler et al., 2000; Baines et al., 2005). The discovery of ferroptosis has come a long way since the 1950s, although it was only named in Dixon et al. (2012). In the following year, the term “autosis” was described by Beth Levine following the observation of a subtype of cell death associated with autophagy induced by nutrient deprivation or Tat-Beclin 1 [one of the peptides inducing autophagy by BECN1 and human immunodeficiency virus (HIV) Tat protein] (Liu et al., 2013). Novel observations regarding neuronal cell death continue to be reported frequently, both refining and redefining known paradigms of cell death, such as apoptosis, necroptosis (Arrazola and Court, 2019), autophagic cell death (Liu and Levine, 2015), ferroptosis (Dixon et al., 2012), and pyroptosis (Fink and Cookson, 2006) (the timeline of the RCDs research is depicted in Figure 1).

All of the discovery in RCDs requires accurate identification techniques, including superficial morphological detection, but the changes in detail are indistinguishable at the morphological level. Biochemical detection, which refers to multiple biomarkers, and functional perspectives based on functional changes, such as assays related to the molecular mechanism of the RCD-related genes (Hengartner and Horvitz, 1994), have widely used flow cytometry in RCDs detection, cytosolic DNA assays, and nucleic acid kits (Boldin et al., 1996; Li et al., 1997, 1998; Luo et al., 1998; Paludan et al., 2019). Various signature proteins involved in cell death have been reported and researchers make use of these proteins in cell death assays. The discovery of the main proteins is shown in the timeline in Figure 1.

Regulated cell death is closely related to physiological and pathological processes, including inflammation, neurodegenerative diseases, immunological diseases, and cancer (Anderton et al., 2020). Therefore, targeting the regulatory mechanisms of RCD is becoming a great opportunity to discover new therapies to target regulated pathways and identify potential drug targets. They can also act as potential targets in diagnosis and prognostic evaluation. Each of the RCDs has a unique molecular mechanism, with special morphological characteristics, and they have established complex connections with each other. Fully understanding their various detection methods, as well as their advantages, is necessary for the efficiency and accuracy of their detection. We have summarized and compared the signaling pathways regulating cell death, mainly including apoptosis, necrosis, autophagy, ferroptosis, pyroptosis, and NETosis, in these aspects: morphology, biochemistry, and function (a brief summary is available in Table 1).

**TABLE 1:** Comparison of five types of RCDs.

Items	Definition	Morphological features	Detecting methods	References
Apoptosis	A vital component of various processes including normal cell turnover, proper development and functioning of the immune system, hormone-dependent atrophy, embryonic development and chemical-induced cell death	Cell shrinkage (pyknosis); DNA fragmentation (karyorrhexis); nuclear condensation; membrane blebbing; apoptotic body formation	DNA fragmentation (DNA ladder assay, TUNEL assay, and comet assay); phosphatidylserine (Annexin V, flow cytometric analysis); bid, p53 (RT-PCR, western blot, immunohistochemistry); caspase activation (western blot, ELISA, flow cytometric analysis); Fas, TNF, TRAIL (RT-PCR, western blot, immunohistochemistry); Cytochrome C release (ELISA)	Saraste and Pulkki, 2000; Hunter et al., 2005; Elmore, 2007; Huerta et al., 2007; Krysko et al., 2008; Majtnerová and Roušar, 2018
Necroptosis	A type of cell death that is caused by the loss of plasma membrane integrity following receptor interacting kinase 3 (RIPK3)-mediated phosphorylation of the pseudokinase mixed lineage kinase domain like (MLKL/pMLKL)	Cell swelling; membrane rupture; retain integral nucleus; translucent cytoplasm	RIPK1, RIPK3, MLKL (immunofluorescent staining); MLKL (quantitative RT-PCR); RIPK1/3, RIP3, MLKL (western blotting); MLKL, RIPK3, RIP1 (ELISA); annexin V–/propidium iodide+ or annexin V+/propidium iodide+ (flow cytometry); RIP1/RIP3 complex (immunoprecipitation and electron microscopy); RIP1 (immunoblotting); membrane translocation (immunofluorescence microscopy and TIRF microscopy);	Aachoui et al., 2013; Chen et al., 2014; He et al., 2016; Gong et al., 2019; Tonnus et al., 2019; Wimmer et al., 2020; Wu Y. et al., 2020
Autophagy	An evolutionarily ancient and highly conserved catabolic process involving the formation of double membraned vesicles called autophagosomes that engulf cellular proteins and organelles for delivery to the lysosome	Lack of chromatin condensation; massive vacuolization of the cytoplasm; accumulation of (double-membraned) autophagic vacuoles; little or no uptake by phagocytic cells	Immune colloidal gold technique; GFP-LC3 or mRFP-GFP-LC3 (immunofluorescence); LC3-II / LC3-I, beclin, ATG5, ATG7, p62 and phosphorylation status of ULK (western blot); LDH sequestration; MDC staining Hsc70 with lysosomal markers (immunofluorescence); LAMP2A (western blot)	Bernocchi and Barni, 1983; Boldin et al., 1996; Broaddus et al., 1996; Boschker and Middelburg, 2002; Brauer, 2003; Brinkmann et al., 2004; Bergsbaken et al., 2009; Burattini and Falcieri, 2013; Braga et al., 2016; Buschhaus et al., 2017, 2018; Bhutia et al., 2019; Yang J. W. et al., 2018
Ferroptosis	An iron-dependent form of regulated cell death caused by unrestricted lipid peroxidation and subsequent membrane damage	The loss of plasma membrane integrity; the leakage of intracellular contents	ATP5G3, PTGS2, IREB2, CS, RPL8 (quantitative real−time PCR); JNK, Erk1/2, p38, LC3I/II, Nrf2, p62, Slc7a11 (western blot); Fe^2+^ release assay; flow cytometry; GPX4 (ELISA); NADP/NADPH, LC3 (fluorescence); immunofluorescence;	Wang H. et al., 2017; Kong et al., 2019; Tang and Kroemer, 2020
Pyroptosis	A form of lytic cell death that is triggered by proinflammatory signals and associated with inflammation	Membranous pore formation; cytoplasmic swelling; rupture of the cell membrane and release of its intra-cellular contents into the immediate cellular milieu	BCA, casp1 (western blot); real-time PCR; caspase-1, CD31 (TUNEL staining and immunostaining); IL-1β, IL-18, pro-IL-1β, IL-1α (ELISA); FAM-FLICA-caspase-1 and PI (flow cytometry); NLRP3, caspase-1, IL-1β, IL-18 (immunofluorescence); Ca^2+^ (fluorescence); IL-1β, casp1, casp8 (immunoblotting); determination of LDH; PLFA (isotope labeling); spectral analysis; DAB, AEC (chromogenic staining)	Boschker and Middelburg, 2002; Schneider et al., 2017; Lei et al., 2018; Wang et al., 2018a; Wu et al., 2018; Yang F. et al., 2018

*ATG5/7: Autophagy-Related protein 5/7, ATP5G3: isoform 3 of subunit c of mitochondrial ATP synthase, CD31: platelet/endothelial cell adhesion molecule-1, ELISA: enzyme linked immunosorbent assay, Erk1/2: extracellular signal-regulated kinase 1, FLICA: fluorochrome-labeled inhibitors of caspases, GFP: green fluorescent protein, GPX4: glutathione peroxidase 4, IREB2: iron-responsive element-binding protein 2, LAMP2A: lysosome-associated membrane protein 2A, LC3: light chain 3, LDH: lactate dehydrogenase, MLKL: mixed lineage kinase domain-like pseudokinase, NLRP3: nod-like receptor protein-3, Nrf2: nuclear factor erythroid 2 p45-related factor 2, PLFA: phospholipid fatty acid, PTGS2: prostaglandin endoperoxide synthase 2, RFP: red fluorescent protein, RIPK1/3: Receptor-interacting serine/threonine-protein kinase 1/3, Slc7a11: solute carrier family 7 member 11, TIRF: total internal reflection fluorescent, TNF: tumor necrosis factor, TRAIL: tumor necrosis factor-related apoptosis-inducing ligand, TUNEL: terminal deoxynucleotidyl transferase (TdT) dUTP Nick-End labeling, ULK: Unc-51-like kinas.*

## Cell Viability Assays

Once cell death is induced, the plasma membrane integrity would be destroyed, or before losing its integrity, the corpse or fragments of the cell would be engulfed by neighboring cells *in vivo*. The cells’ contents can also be exposed or even lost (e.g., the spillage of cytosolic lactate dehydrogenase (LDH), the exposure of DNA), the activity of intracellular enzymes could decline (e.g., succinate dehydrogenase), and a reduction in intracellular adenosine triphosphate (ATP) is observed, which reflects the cellular energy capacity and viability. In view of these phenomena, spectrophotometry, fluorometry, flow cytometry, and microscopy are utilized for cell viability assays.

### Spectrophotometry

Spectrophotometry is a qualitative or quantitative analysis of signal material through the absorption of a certain range of wavelengths. Using spectrophotometry to monitor the metabolic activity of cells is regarded as an indicator of cell viability, such as succinate dehydrogenase (SDH) activity analysis, the cytosolic LDH release analysis, certain proteins, and DNA binding by crystal violet staining. The principle of these technologies, in summary, is that the light absorption value of certain markers can be measured by a microplate enzyme for a qualitative or quantitative analysis of cell viability.

Some limitations of SDH activity analysis are as follows: (1) In dead cells, SDH is often still partially active, resulting in being erroneously scored as living cells when it comes to the early stages of apoptosis. (2) Not only cell death, but also other physical and chemical factors can lead to a decrease of enzyme activity, which leads to a false-positive or false-negative result. (3) The activity of an enzyme is variant in different tissue or cells, such as a lesser degree of SDH activity being present in neural and hepatic tissues, which easily leads to errors (Bernocchi and Barni, 1983). (4) The process of MTT conversion is also affected by various conditions, such as the cellular confluency and the culture medium exhaustion, which could lead to under-estimating the cell viability. (5) Some other solutes might elevate background signals by absorbing the same range of wavelengths, such as media containing phenol red, which leads to an increased cell viability range in the assay. All in all, SDH activity analyses of cell viability are usually used in conjunction with other techniques to improve their accuracy.

Crystal violet staining binding by certain proteins and DNA is suitable for monitoring the effect of various compounds (e.g., chemotherapeutics) on cell growth or survival as a reliable and quick screening measure (Geserick et al., 2009). However, some of the special cases would be potentially compromised due to the changed adherent properties of cells, such as the proliferative responses that occur in conjunction with cell death responses or the steps of repeated washing, which might sometimes lead to detached living cells, causing artifacts, especially when there is a higher cell density (Feoktistova et al., 2016).

As for the cytosolic LDH release analysis, some caution should be exercised regarding the instability and proteolytic degradation of LDH activity, since some of the factors may interfere with the results, such as the time spent, the pH, and the specific components present in the culture medium (e.g., pyruvate) (Kendig and Tarloff, 2007).

### Fluorometry, Flow Cytometry, and Microscopy Assay

The plasma membrane integrity is followed by the exposure of intracellular contents, and the dye could enter and bind to these contents in the dead cell. For identifying leaking cell contents and lost membrane integrity, researchers often use a targeted approach for detection, such as light microscopy to detect trypan blue positive cells, and fluorometry and flow cytometry are used for identifying leaking cell contents. The detailed descriptions of each of these technologies is shown below.

Using technologies such as flow cytometry and light microscopy, the dead cells are indicated indirectly. For example, in DNA-binding, impermeant fluorescent dyes are used for detection, such as propidium iodide (PI), 7-amino actinomycin D (7-AAD) or the Sytox probes (Life Technologies) (Riccardi and Nicoletti, 2006; Zembruski et al., 2012, Thakur et al., 2015). For instance, during apoptosis, PI is able to bind and label DNA fragments and makes it possible to provide a rapid (in about 2 h) and precise method for evaluating intracellular DNA fragments through flow cytometry, and subsequently identifying the hypodiploid cells (Riccardi and Nicoletti, 2006). Compared with crystal violet staining analysis by spectrophotometry as mentioned above, this reduces the steps used to wash cells.

Notably, trypan blue staining could detect all forms of cell death, but differentiating among the specific types of cell death needs further testing (Crowley et al., 2016a). Moreover, other assays for detecting the loss of plasma membrane integrity have been adapted, such as immunocytochemistry to assay protein translocation, and 4′,6′-diamidino-2-phenylindole (DAPI) staining to assay nuclear fragmentation (Crowley et al., 2016b). For example, immunocytochemistry, such as for cytochrome c, is regarded as an essential tool for understanding and characterizing the mitochondrial apoptosis pathway (Crowley et al., 2016c).

## Determining Cell Morphology of RCDS

Incipiently, differentiating RCDs usually relied on morphological changes limited by techniques while the three major types of cell death are identified (type I cell death refers to apoptosis, type II cell death corresponds to autophagy-dependent cell death, type III cell death is related to necrosis) (Kerr, 1971; Lin et al., 1973). In Lockshin and Williams (1964) the American biologist Richard A Lockshin thoroughly described PCD (Lockshin and Williams, 1965), and in Kerr et al. (1972), the Australian pathologist John F Kerr and his colleagues coined the term “apoptosis.” They analyzed RCDs based on morphological changes. Later, the Nomenclature Committee on Cell Death (NCCD) updated the classification system of cell death through more comprehensive aspects, including classification (Kroemer et al., 2005, 2009), molecular definitions (Galluzzi et al., 2012), essential vs. accessory terms (Galluzzi et al., 2015), and molecular mechanisms (Paredes-Gamero et al., 2012). Meanwhile, the technology of RCDs detection is developing rapidly. To date, these detection techniques have been developed for monitoring the morphology of RCDs, such as light microscopy (Paredes-Gamero et al., 2012), EM, transmission electron microscopy (TEM) (ultrastructural changes and chromatin condensation in the cells) (Dong et al., 2015), scanning electron microscopy (surface changes of cells or tissues) (Burattini and Falcieri, 2013), atomic force microscopy (whole-process changes in RCDs) (Kuznetsov et al., 1997; Hessler et al., 2005; Sborgi et al., 2016), fluorescence microscopy (FM) (specific fluorescence labeling such as NAD(P)H-labeled in apoptosis) (Alturkistany et al., 2019), and practical flow cytometry (number and rate of dead cells) (Yasuhara et al., 2003; Paredes-Gamero et al., 2012; Liao S. et al., 2019).

**FIGURE 2:**
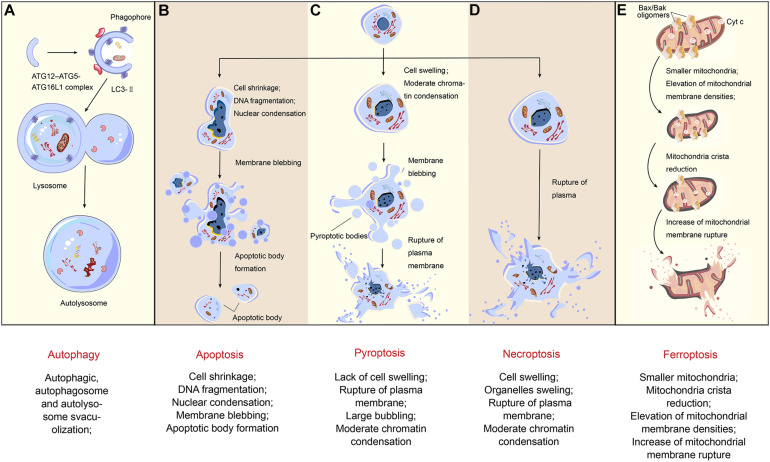
Morphological changes of regulated cell death. Different types of cell death inducing with various pathways present diverse morphological changes. **(A)** The formation of autophagosomes is the typical characteristic of autophagy. ATG12-ATG5-ATG16L1 complex and LC3-II contribute to the extension of the phagophore. When the autophagosome is completely formed, LC3-II will separate from the outer membrane, and the autophagosome fuses with the lysosome so that an autolysosome finally emerged. **(B)** As for apoptosis, in the early stage, the nuclear condensation starts with cell shrinkage. During later stages, nucleus breaks up and the plasma membrane bubbles with no rupture, naturally associating with no inflammation. Finally, it forms apoptotic bodies. **(C)** Being different from apoptosis, after cell swelling and large bubbling, pyroptosis has plasma membrane rupture in the final stage. **(D)** When it comes to necroptosis, the appearance of cell swelling often followed by organelles dilation, and the nucleus disintegrates late. In some cases, chromatin condensation also occurs. Finally, with the rupture of plasma, a massive inflammation in the tissue is triggered. **(E)** The morphological characteristics of ferroptosis, however, mainly reflects on the changes of mitochondria. Compared with organelles swelling in necroptosis, here in the first stage, the mitochondria become smaller and membrane densities are elevated, followed by mitochondria crista reduction and plasma membrane rupture.

As the most intuitive means, morphologic detection also refers to many different aspects: the alterations of the membrane (e.g., the loss of membrane integrity), the changes in cytoplasmic contents (e.g., mitochondrial damage), and the alterations of the nucleus and DNA. Each of the RCDs has their own iconic characteristic presented through immunohistochemistry (IHC) or various fluorescent dyes and high-resolution microscopy (Figure 2).

### Time-Lapse Microscopic Analysis

Time-lapse microscopic analysis, involving a temperature and CO_2_-controlled incubator, has benefits for monitoring a biological process over a time period, which may last from a few hours to several days (Wallberg et al., 2016b). It can be suitable for a comparative and dynamic study of single cells *in vivo* or in culture shown as automated imaging of visible cell surface changes. This technique could be recorded *via* using differential interference contrast (DIC) optics either alone or in combination with epifluorescence microscopy specifying a predefined delay between the acquisition of images. The related parts of this technology may be changed slightly, generally including a Leica ASMDW live cell imaging system (Leica Microsystems, Mannheim, Germany), which includes a DM IRE2 microscope equipped with an HCX PL APO 63/1.3 glycerin corrected 37°C objective and a 12 bit Coolsnap HQ Camera (Cappellini et al., 2005; Krysko et al., 2008; Chan et al., 2011).

After staining with combinations of dyes, cell death can be visualized. These dyes include Alexa Fluor 647-conjugated Annexin V and Sytox Green (SG), or Annexin VFITC and PI. Recently, fluorescent probes have been developed to improve the accuracy during detection, such as two-related fluorescent probes, namely molecular conjugates of one or two zinc dipicolylamine (ZnDPA) coordination complexes with an appended solvatochromic benzothiazolium squaraine dye targeting the anionic phospholipids and phosphatidylserine (PS) exposed on the surface of dead or dying cells (Jarvis et al., 2018). This method is considered the best choice to distinguish between necrosis and apoptosis morphologically (Wallberg et al., 2016b). Annexin V staining using fluorescein isothiocyanate (FITC) could be used to detect apoptosis through binding to PS in the presence of Ca^2+^ (Vermes et al., 1995; Baskic et al., 2006). In necrosis, Annexin V staining can present a positive, so researchers always use double staining of Annexin V and PI to confirm necrosis (Vermes et al., 1995), and it has been proven that ^99m^Tc-radiolabeled Annexin V is the most successful marker (Brauer, 2003).

Obtaining the data of cell morphology in apoptosis is described as: roundness, blebbing, the breaking up of cell fragments into apoptotic bodies (a process called “budding”), which are eventually degraded within phagolysosomes, and nuclear condensation (karyopyknosis) and fragmentation (karyorrhexis), and it always happens in small clusters of cells or individual cells without inflammation. After that, apoptotic cells are consumed via phagocytic systems *in vivo*, or eventually lose the integrity of their plasma membrane and undergo secondary necrosis, which is characterized by plasma membrane permeabilization and osmotic swelling without phagocytosis. As for necroptosis, cellular swelling and a balloon-like-structure formation (“oncosis”) are regarded as the major characteristic changes with inflammation.

However, it can also be challenging to distinguish apoptosis from necroptosis because undergoing secondary necrosis might occur in apoptotic cells where Annexin V-binding can enter cells through a broken membrane. To avoid this case, the combination of Annexin V and noncell permeable DNA stains (e.g., PI or SG) is adopted (Wallberg et al., 2016b; Jarvis et al., 2018).

Time-lapse microscopic assays can also be used to show some of the specific molecular markers, such as DNA, RNA or proteins involved in their molecular pathways and functions through using fluorescent molecular probes, such as monitoring autophagy *via* time-lapse microscopy to track Parkin (a protein implicated in mitophagy) fused with fluorescent enhanced yellow fluorescent protein (EYFP) (Di Sante et al., 2016). This method has advantages for collecting information at the single-cell level. However, assays based on time-lapse microscopy are susceptible to some factors (e.g., vibrations, the fluctuations of temperature and humidity, pH and cell motility, etc.) that may interfere with the acquisition of high-quality images (Di Sante et al., 2016).

### EM Analysis

Electron microscopy analysis has always supplied us with a great deal of data in cell death research, in which TEM benefits from its much higher resolving power (0.1-0.4 nm) and scanning electron microscope (SEM) also offers high resolving power (about 1 nm). TEM makes it possible to better understand the relationships between a biological structure and its function at the cellular, subcellular, and even molecular levels through two- and three-dimensional images of the cells to distinguish different forms of RCDs, which is considered as a “golden standard” (Ericsson, 1969; Krysko et al., 2008). SEM, a kind of observation at the level between TEM and the light microscope, could emerge as a good method to provide a delicate imaging stereo effect of the surface of the cells with a magnification of 300,000 times or more. SEM is utilized for topographic imaging of bulk samples suited for low energy EM, whose maximum electron energies are at 30 keV (Sun et al., 2018). In addition, SEM combined with other analytical instruments can be used to observe the microscopic morphology and to carry out a composition analysis of the material microregion. However, many researchers consider EM analysis to be time-consuming and expensive.

Meanwhile, the preliminary substrate preparation of dying cells for EM could be difficult during the intermediate process because it may cause damage to the original morphology of the RCDs, especially when they detach the dying cells from their substrate, typically resulting in spinning down these floating cells. Recently, EM has been optimized continuously; for example, using macrophages or cytospinning to capture the cells prevents interference with their cell morphology (Vanden Berghe et al., 2013; Crowley et al., 2016d).

SEM/STEM/TED imaging can capture a wealth of information and focused ion beam scanning electron microscopy (FIB/SEM) (Knott et al., 2011), one of the three-dimensional EMs, is being increasingly adopted in life sciences. It is worth noting that the discovery of cryo-electron microscopy (cryo-EM), leading to a detailed or realistic display of ultrastructure in cells, may provide more convincing evidence in RCDs (Li et al., 2018; Fitzpatrick and Saibil, 2019).

#### Determining Cell Morphology in Apoptosis

As for apoptosis, people have marked it by the typical morphological changes: cell shrinkage, DNA fragmentation (karyorrhexis), chromatin changes during condensation and margination, a ruffling plasma membrane, and breaking up of cell fragments into apoptotic bodies (a process called “budding”), which are eventually degraded within phagolysosomes. Previously, light microscopy has identified the morphological changes occurring during apoptosis with cellular shrinking and pyknosis (Kerr et al., 1972). After hematoxylin and eosin stain (HE staining), the histologic characteristics of apoptosis are defined as confirming a round or oval mass, a red-stained eosinophilic cytoplasm, and purple-dense nuclear chromatin fragments, usually occurring in small clusters of cells or individual cells without inflammation. Besides, the more detailed morphological changes have been confirmed by EM, which could better present the subcellular changes, such as the most characteristic feature of apoptosis, pyknosis in which uniformly dense masses of chromatin are formed and distributed against the nuclear envelope after chromatin condensation (Hacker, 2000; Zucker et al., 2000). Additionally, the loss of cell-to-cell contacts and microvilli are visible in the images (Kerr et al., 1994; Cummings et al., 1997).

#### Determining Cell Morphology in Necroptosis

Compared to apoptosis, necrosis emerges as a general swollen cell (oncosis) and the swelling of the cytoplasmic organelles (e.g., swollen mitochondria), poorly demarcated clumps of chromatin, a rapid loss of plasma membrane integrity, and the release of cytoplasmic contents, which is uncontrolled and passive, and eventually, the changes of nuclear morphology are described as pyknosis, karyorhexis, and karyolysis. In detail, the major morphological changes during necrosis could be summarized as: (1) nucleus: pyknosis for the nuclear dehydration, karyorhexis (nuclear fragmentation), karyolysis (without the visible outlines of the nuclei); (2) cytoplasm: cytoplasmic vacuoles forming; endoplasmic reticulum swelling; cytoplasmic blebs forming; mitochondria condensing, swelling or breaking up; ribosomes disaggregating and rupturing; (3) others: cellular swelling; membranes disrupting; eventually; an inflammatory response occurring (Trump et al., 1997). Necrosis is another form of cell death that has some similar aspects to apoptosis, due to several processes like the morphologies and mechanisms shared between them (Kerr et al., 1972; Zeiss, 2003). The characteristic morphology in necrotic cells, such as cytoplasmic swelling, is due to the membrane permeabilization being an early event. Losing membrane permeability in apoptotic cells occurs relatively late.

#### Determining Cell Morphology in Autophagy

Acting as a non-invasive response to cell death with the formation of large-scale autophagic cavitation, autophagy contributes to maintaining the balance of the cellular structure, metabolism, and biological function for homeostasis (Deter and De Duve, 1967; Mizushima and Komatsu, 2011). There are three types of autophagy according to the pathways in which cargoes or phagocytes are transported into lysosomes: microautophagy, macroautophagy, and chaperone-mediated autophagy (CMA) (Rogoza et al., 2004). A comparison of these three forms of autophagy is shown in Table 2. Remarkably, the application of TEM has promoted the progression of autophagy from the phagolysosome to related protein imaging using the cryo-EM structure (Ohsumi, 2014; Hurley and Nogales, 2016).

**TABLE 2:** Comparison of distinguishing criteria, morphological features and monitoring methods of three kinds of autophagy.

Items	Definition	Morphological features	Detecting methods	References
Microautophagy	Cytoplasmic contents are transported to the lysosome within lysosomal membrane invagination or deformation.	Lysosomal membrane invagination or deformation	Transmission electron microscopy	Mijaljica et al., 2011; Parzych and Klionsky, 2014
Macroautophagy	Cargo is transported to the lysosome by de novo formation of autophagosomes.	Membrane expansion and bend; phagophore nucleation and elongation; autophagosome formation	Electron microscopy; immune colloidal gold technique; GFP-LC3 or mRFP-GFP-LC3 (Immunofluorescence); LC3-II / LC3-I, Beclin, ATG5, ATG7, p62 and phosphorylation status of ULK (western blot); radiolabeling; LDH sequestration; MDC staining	Parzych and Klionsky, 2014; Seglen et al., 2015; Murugan and Amaravadi, 2016; Orhon and Reggiori, 2017; du Toit et al., 2018; Yang F. et al., 2018
CMA	Unfolded proteins containing the KFERQ motif are transported directly across the lysosomal membrane through the action of cytosolic chaperones.	Multimerization of LAMP2A binding to the lumenal side of the lysosomal membrane by HSP90	Hsc70 with lysosomal markers (Immunofluorescence); LAMP2A (western blot); radiolabeling	Cuervo, 2010; Parzych and Klionsky, 2014; Patel and Cuervo, 2015; Arias, 2017; Wu et al., 2019

*Abbreviations: LAMP2A: lysosome-associated membrane protein 2A, HSP90: Heat Shock Protein 90, ATG5: Autophagy-Related Gene 5, ATG7: Autophagy-Related Gene 7, Hsc70: heatshockprotein.*

##### Macroautophagy

Among the three types of autophagy, macroautophagy has been studied extensively and clearly. It is induced under stress stimulus, resulting in maintaining cell growth through specifically degrading damaged or superfluous organelles or leading to various human pathologies, including lung, heart, neuron, and liver disease, cancer, myopathies, aging and so on, after excessive self-degradation (Yorimitsu and Klionsky, 2005; Parzych and Klionsky, 2014; Pastuhov et al., 2016). Morphologically, the autophagosome is one of the distinct features forming by expansion as well as *de novo* rather than membrane budding from an already contained cargo (du Toit et al., 2018). The membrane expands and bends, and then a spherical autophagosome is generated. Using electron microscopic examination of the obverse, it has been found that the diameter of autophagosomes ranges from 0.5 to 1.5 μm in mammals and ∼0.4 to 0.9 μm in yeast (Takeshige et al., 1992; Gu et al., 2020). Besides autophagosomes, autophagy precursors (which include the cytoplasm, organelles, or bacteria) and autophagolysosomes (autophagosomes that bind to the lysosome) are also vital subcellular structures.

##### Microautophagy

For microautophagy, most of the research has focused on yeast and describes its transport pathway as cytoplasmic contents are transported into the lysosome within the lysosomal membrane invagination (Marzella et al., 1981). De Duve *et al.* first observed microbodies in autophagy using EM (Baudhuin, 1966). Also, Sakai et al. observed the process of microautophagy using vacuoles and microbodies of yeast with fluorescent double-labeling (Itoh et al., 1998). Then, nucleus microautophagy in yeast was reported by Roberts *et al.* (Fernandez et al., 2003). Early on, using EM, people noticed the free-floating vesicles within the lysosome through englobing Percoll particles by way of cup-like invaginations of the lysosomal membrane (Marzella et al., 1980). Recently, nucleophagy, which targets autophagic degradation as nuclear material, has helped in elucidating the endosomal microautophagy transports detected by indirect immunofluorescence techniques (Otto and Thumm, 2020). Microautophagy is so small that TEM is needed to see it clearly. Due to the limitations of technology, we know little about microautophagy, including the inducing factors, the mechanism, and its role in the disease processes (Mijaljica et al., 2011; Sato et al., 2019; De Falco et al., 2020).

##### Chaperone-mediated autophagy

Compared with microautophagy and macroautophagy, one of the nonspecific pathways to engulf the cytoplasm, CMA, is highly specific and refers to a specific substrate (a compound formed by a pentapeptide sequence that is biochemically related to KFERQ, namely Lys-Phe-Glu-Arg-Gln and HSPA8/HSC7, the heat shock 70 kDa protein 8) and specific receptor LAMP2A, the lysosomal-associated membrane protein 2A (Chiang et al., 1989; Dice, 1990). The morphological changes of CMA are not obvious, so immunofluorescence analyses, western blots, and other biochemical methods play crucial roles in CMA assays (Wu et al., 2019).

##### Non-canonical autophagy

Besides these classical autophagy pathways, the recent identification of non-canonical autophagy pathways, such as LC3-associated phagocytosis (LAP) and LC3-associated endocytosis are showing impact on cell viability (Martinez et al., 2016). This non-canonical autophagic process relies on Rubicon (rubicon autophagy regulator [RUBCN]), contributes to immunosuppression (Sil et al., 2020). And these specific pathways have been reported to underly the pathogenesis of various diseases. For example, LAP is closely related to systemic lupus erythematosus (SLE) (Bandyopadhyay and Overholtzer, 2016; Martinez et al., 2016), tumor (Cunha et al., 2018). And LC3-associated endocytosis has been shown to be protective against neuronal cell death in Alzheimer’s Disease (Heckmann et al., 2020a, b). Similar to canonical autophagy, LAP require ATG-7/-3/-5/-12/-16L for LC3 lipidation (Martinez et al., 2011, 2015). But unlike autophagy, LAP is a process requiring NADPH oxidase-2 (NOX2)5, and Rubicon5 (Martinez et al., 2015), and LAP is independent of the pre-initiation complex containing ULK1 and FIP200 and proceeds with a distinct Beclin 1-VPS34 complex lacking ATG14 (Florey et al., 2011; Martinez et al., 2011; Kim et al., 2013).

All in all, these three canonical autophagy pathways could deliver the cargo to the lysosome to degrade and recycle while each of them features with different morphology. The formation of autophagosome is regarded as characteristic changes during macroautophagy, which is formed by portions of the cytosol and intact organelles (e.g., mitochondria) sequestered into a double-membrane vesicle (Mijaljica et al., 2011). By contrast, microautophagy involves the direct engulfment of cytoplasm at the lysosome surface requiring the EM for detecting, whereas CMA refers to a process delivering soluble and unfolded proteins directly across the limiting membrane of the lysosome (Massey et al., 2004).

#### Determining Cell Morphology in Ferroptosis

As an oxidative, iron-dependent form of RCD, ferroptosis is induced through an excess accumulation of reactive oxygen species (ROS) and lipid peroxidation products (Dixon et al., 2012; Chen et al., 2020). When undergoing ferroptosis, dysmorphic small mitochondria or mitochondria shrinkage with enlarged and reduced crista, and a condensed and ruptured membrane can be detected morphologically using an electron microscope (Dixon et al., 2012; Friedmann Angeli et al., 2014; Doll et al., 2017). Although relevant research in ferroptosis has made rapid progress, using mitochondrial morphology to distinguish ferroptosis is still highly debatable since there is a lack of studies on the correlation between mitochondria and ferroptosis (Dixon et al., 2012; Gaschler et al., 2018).

#### Determining Cell Morphology in Pyroptosis

Pyroptosis refers to a process in which the pores on plasma membranes are gradually formed, inflammatory cytokines are released, and the lysed cells are induced through the canonical caspase-1-mediated monocyte death or the non-canonical caspase-4/5/11 inflammasome pathways (He et al., 2019; Wang et al., 2019e). The morphological changes during pyroptosis include cell swelling, which is induced through entering water molecules, the formation of 10-15 nm pores in the plasma membrane, and the eventual release of pro-inflammatory cytokines (interleukin-1β (IL-1β) and interleukin-18 (IL-18)), distinct from apoptosis without inflammatory release (Lu et al., 2020). Activating caspases, an N-terminal cleavage product (GSDMD-NT) would be generated, which triggers inflammatory death (pyroptosis) and the release of inflammatory cytokines such as IL-1β 1,2 (Shi et al., 2015). Notably, it is visible by EM since GSDMD-NT oligomerizes in membranes to form pores to trigger pyroptosis (Liu et al., 2016). These morphological changes have been observed in smooth muscle cells (SMC), endothelial cells (EC), macrophages, phagocytes, astrocytes and neurons, and additional research on its mechanism and technical studies are needed in the future (Liu et al., 2018, 2020).

#### Determining Cell Morphology in NETosis

NETosis, one of the RCD types which was first described in 2004, is regarded as a program for formation of neutrophil extracellular traps (NETs) initiating a fight against pathogens and linking to various diseases (Brinkmann et al., 2004). According to the viability of cells, NETosis could be divided into two different forms, namely classical or suicidal NETosis resulting in the cell death, and vital NETosis retaining viability. The typical morphological changes of classical NETosis are described as chromatin decondensation associated with histone modification, and the release of granule components into the cytosol, as well as many characteristic features which are the same as other forms of RCDs (such as the changes in the nucleus and in the cytoplasm during apoptosis, necroptosis, pyroptosis, and autophagy (Vorobjeva and Chernyak, 2020). As for vital NETosis, it refers to a massive and very fast release of mitochondrial DNA (mtDNA) without loss of viability, when neutrophils retain their viability and natural effector functions (Yousefi et al., 2008, 2009).

### Flow Cytometry Assay

Flow cytometry is used as a general measure for rapid analysis of a large number of cells individually, which detects up to 10,000 cells per second. Based on the cellular characteristics, including the size, granularity and morphology of the cells, the integrity or potential of the cell membrane, the intracellular pH, and the levels of cellular contents such as surface receptors, proteins, the ions (e.g., calcium), DNA, and RNA, flow cytometry could provide data to distinguish RCDs through the use of fluorescence, absorbance measurements, and light-scattering.

Currently, flow cytometry is used not only in cell counting but also in image analysis, namely multispectral imaging cytometry for multiparameter studies on cell demise (Lelliott et al., 2019; Cerrato et al., 2020). Morphology-based flow cytometry assays are used to identify RCDs, such as the percentage of apoptotic cells that are indicated based on the cells exhibiting nuclear fragmentation as well as a low nuclear area and a bright detail intensity. Various staining approaches such as 1 μM camptothecin (CPT; Sigma, a DNA topoisomerase I inhibitor) for 6 hours, fixed and stained with PI, and collected on the ImageStream (George et al., 2004), can be applied. Moreover, cells undergoing autophagy can be identified through visualizing fluorescently labeled lysosomal markers, and LC3 puncta labeled and/or the co-localization of fluorescently labeled LC3 using flow cytometry provide benefit in an objective, quantitative, and statistically robust manner (Pugsley, 2017). Also, the loss of plasma membrane integrity using cell-impermeant dyes could be assessed through a morphology-based flow cytometry assay to detect cell viability (Vanden Berghe et al., 2013). Furthermore, flow cytometry is often used to monitor RCDs with biomarkers and we will discuss this below.

### Confocal Laser Scanning Microscopy

Detection work needs a point scanning confocal microscope with good optical efficiency. Importantly, a good lens with a long working distance and high NA determines the rendering effect. The Leica TCS4D or SP1 with a Leica inverted IRMB microscope and an Argon-Krypton laser (Omnichrome, Chino, CA, United States) emitting 3 wavelengths (488, 568, and 647 nm) have been used in confocal microscopy (Zucker and Rogers, 2019). A 5 × or 10 × objective with a high numerical aperture (NA) and the lenses including Zeiss 5 × fluor (NA 0.25), Zeiss 10 × fluor (NA 0.5), Leica 10 × Plan APO (NA 0.5), and Leica multi-immersion 10 × (NA 0.4), also a Zeiss lens fitting on a Leica microscope whose magnification could be increased by 20%, are available (Zubairova et al., 2019; Zucker and Rogers, 2019). Confocal microscopy could provide a visible three-dimensional structure as a good indicator for RCDs visualization, and it is also flexible and fast except for some cumbersome processes, namely staining, fixation, dehydration, and clearing (Zucker and Rogers, 2019). For example, confocal microscopy is used to provide insights into the dynamics of cell death with the fluorescent dyes fluorescein diacetate (FDA) and PI (Jones et al., 2016). Also, the research into mitophagy using confocal microscopy and the subcellular localization of ceramide in mitochondria were visualized by colocalization of ceramides and mitochondria (Sentelle et al., 2012).

### FM

Fluorescence microscopy is often utilized to monitor RCDs *via* not only detecting specific markers, but also for evaluating cell nucleus damage and DNA, leading to direct visualization of pathophysiological processes with sub-cellular resolution. On this basis, FM is constantly being improved for various requirements in detection, such as fluorescence lifetime imaging microscopy (FLIM) being used to monitor caspase-3 activity during apoptosis (Buschhaus et al., 2017, 2018). Moreover, autolysosomes for autophagy detection can be visible via the colocalization of LC3 and lysosomal markers by FM (Yoshii and Mizushima, 2017; Bhutia et al., 2019). Caspase-1 activity assays for pyroptosis detection could also be adapted to FM, such as with a Nikon ECLIPSE TE2000-U FM in Tokyo, Japan (Liao Y. et al., 2019). The changes of the nuclei could therefore be identified through FM to distinguish apoptotic cells from healthy cells or necrotic cells for staining with DAPI or other dyes (Crowley et al., 2016). DAPI or Hoechst 33258, 33342, and 34580 are often used to monitor DNA fragmentation or damaged cell nuclei through binding to A-T base pairs lining the minor groove of double-stranded DNA (Eriksson et al., 1993; Martin et al., 2005). FM has tended to be artificially intelligent, and its probes have become smaller and more prominent with more precise targeting options.

### Intravital Multiphoton Microscopy

People also pay more attention to RCDs monitoring *in vivo*. Intravital multiphoton microscopy has been developed in the cell death process for monitoring at the cell level in tissues *in vivo* (Mesa et al., 2015). It is an objective and real-time technique for capturing morphological changes during RCDs (e.g., apoptosis characteristics with membrane blebbing and ApoBD formation) *in vivo* with a fluorescence resonance energy transfer (FRET)-based caspase 3 activation reporter (Garrod et al., 2012; Mayer et al., 2017). For staining, poly ((3-((4-methylthiophen-3-yl)oxy)propyl) triphenylphosphonium chloride) (PMTPP), one of the fluorescent sensors or stains used for monitoring ATP levels in cell membranes to monitor cell death processed *in vivo* through FM, is available (Huang et al., 2017). To make the detection in RCDs more realistic, standard, noninvasive, clinical, magnetic resonance imaging and spectroscopy (MRI/MRS), computed tomography (CT), positron emission tomography (PET), and radionuclide imaging methods are also used for monitoring the biochemical and physiological processes in apoptosis, necrosis, autophagy, and ferroptosis (Brauer, 2003; Lee et al., 2019).

## Monitoring and Measuring RCDs by Biomarkers

To make the identification of RCDs more objective and quantitative, researchers always combine morphological changes and biomarkers of RCDs. RCDs have different pathways involved with specific proteins; for example, caspase-3 is an important effector of apoptosis, and once activated, it leads to apoptosis (Li X. et al., 2019). Although detection of biomarkers makes RCDs identification more accurate, it can be challenging to distinguish different forms of RCDs clearly for the discovery of new RCDs mechanisms and interlaced protein pathways.

**FIGURE 3:**
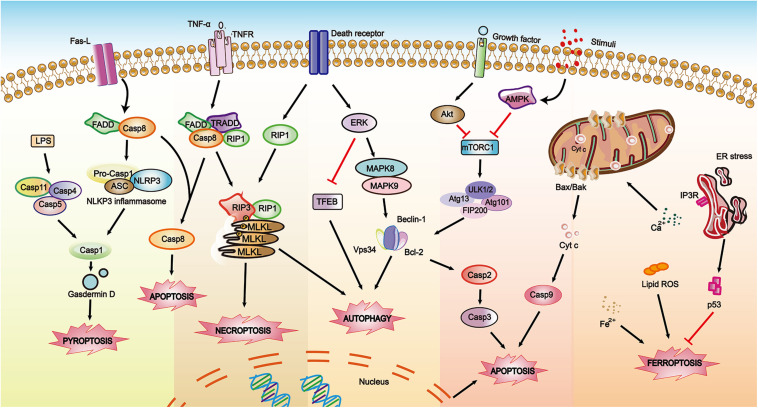
A schematic diagram showing biomarkers involved in RCDs within depicting the cross-regulations among different pathways. The solid arrows indicate activating interactions while the T-shaped lines indicate inhibitory interactions.

Depending on where the content exists and how the content is extracted, the molecular biomarkers used in RCDs detection are divided into three types: cell surface markers acting at the plasma membrane (e.g., PS (PtdSer), pannexin 1 (PANX1)), intracellular markers working inside the cells (e.g., caspase activity, mitochondrial potential), and soluble extracellular markers as well as released molecules with a potential role as circulating biomarkers of cell death (e.g., caspase-3, cytokeratin 18 (CK18), HMGB1 and the enzyme LDH) (Krysko et al., 2008; Hashimoto et al., 2016; Shukuya et al., 2020; Wimmer et al., 2020). The conventional biomarkers of RCDs are shown in Table 3. These vital biomarkers are shown in a schematic diagram (Figure 3), which depicts the cross-regulation among different types of cell death.

**TABLE 3:** Conventional biomarkers for RCDs.

Species of RCDs	Biomarker	Research Model	Death induction	Detecting methods	Expected results	Advantages	Disadvantages	References
Necrosis	Phosphorylation Status of RIPK3, RIPK1 and MLKL	Mice of oligodendrocyte degeneration	N/A	Immunohistochemistry	Upregulated	It is ideal for detecting necrosis in vivo	Most of the antibodies are only suitable for immunoblotting	He et al., 2016
	LDH	Cortical neurons	0.25, 0.5, 1, 2, 3 mM METH and 39°C	Cytotoxicity assay	Upregulated	N/A	It might be influenced by the effect of other species of RCDs	Guo et al., 2020
	Annexin V (FITC)	HT1080^*indRIPK3*^ cells and L929 cells	10 ng/mL TNF, 500 nM SM	Fluorescence-activated cell sorting	Annexin V^*FITC*^, DAPI Upregulated and TMRM Downregulated	It is a fast and quantitative method to record the number of dying cells	It should be combined with a time-course study, and/or the use of specific inhibitors	Wallberg et al., 2016a
Apoptosis	Annexin V (FITC)	HT1080^*indRIPK3*^ cells and L929 cells	10 ng/mL TNF, 500 nM SM	Fluorescence-activated cell sorting	Annexin V^*FITC*^ Upregulated and TMRM, DAPI Downregulated	It is a fast and quantitative method to record the number of dying cells	It should be combined with a time-course study, and/or the use of specific inhibitors	Wallberg et al., 2016a
	Caspase3	Platelet	N/A	Flow cytometric analysis and immunofluorescence	Upregulated	It is a specific method for the active form of caspase-3	N/A	Gyulkhandanyan et al., 2012
	PDK1, P-AKT1, BAD, Bcl-2, Bax	C2C12 cells	0.05, 0.5, 1, 2.5, 5- and 10- mM Fluoride	Real-time PCR and western blot	PDK1, P-AKT1 Downregulated and BAD, Bcl-2, Bax Upregulated	N/A	N/A	Zhou et al., 2018
	DNA fragmentation	NIH-3T3 cell line	500 μM Hydrogen peroxide	DNA ladder assay	DNA ladder formed	It is an easily available method and not limited to cells that breed *in vitr*o	It is a time-consuming procedure	Rahbar Saadat et al., 2015
Autophagy	GFP-LC3 or mRFP-GFP-LC3 Probe	Zebrafish	Rapamycin or Calpain Inhibitors	Immunofluorescence	Being yellow under neutral pH conditions, and being red under acidic pH conditions	It allows to examining autophagy *in vivo* in vertebrates	It might be influenced by the impairment of autolysosome formation.	Lopez et al., 2018
	GFP-LC3-RFP-LC3ΔG probe	GFP-LC3-RFP-LC3ΔG mice	Starvation	Immunofluorescence	GFP/RFP ratio Downregulated	It can monitor both basal and induced autophagy accurately	It is limited by the level of GFP-LC3-RFP-LC3ΔG currently.	Yoshii and Mizushima, 2017
	LC3-II/p62	Exosome in advanced soild tumor patients’plasma	CQ or HCQ	Western blot	LC3-II/p62 ratio Upregulated	It can monitor the dynamic changes on autophagic activity	It might be influenced by the effect of apoptosis and necrocytosis	Abdel Karim et al., 2019
	Autophagosome	HCT-116 colon cells	100 nM Rapamycin or overexpression of Beclin1	Immunofluorescence (MDC Staining)	Fluorescence emitted	MDC staining is readily to operate and directly visualize autophagosome formation	MDC staining is non-specific	Yang F. et al., 2018
Pyroptosis	IL-1β, IL-18	Wistar rats and rat chondrocytes	40 mg/mL MIA in 0.9% NaCl solution, 50 μL	Real-time PCR, western blot and enzyme-linked immunosorbent assay	Upregulated	This assay is highly sensitive	Enzyme activity is easily affected and this assay takes many complex measurements	Zu et al., 2019
	NLRP3, caspase-1	Wistar rats and rat chondrocytes	40 mg/mL MIA in 0.9% NaCl solution, 50 μL	Real-time PCR, western blot, flow cytometry, immunofluorescence and immunohistochemistry	Upregulated	This assay is highly sensitive	These multiple steps are time-consuming	Zu et al., 2019
Ferroptosis	ACSL4	HepG2, HL60, LNCaP, and K562 cells	2.5, 5, 10,20 μM Erastin	Western blot	Upregulated	N/A	N/A	Yuan et al., 2016

### Biomarker Detection Technology

#### Western Blot

The main measures for biomarker detection can be considered at three levels, namely DNA, RNA, and proteins. As for gene/mRNA/cfDNA assays, quantitative PCR (qPCR) is a useful and convenient technique. For protein detection, western blots are regarded as a classic technique. The western blot allows for specific identification and characterization of proteins, able to detect specific proteins involved in RCDs to distinguish RCDs. The process is as follows: the proteins are separated through sodium dodecyl sulfate-polyacrylamide gel electrophoresis (SDS-PAGE), then the polyvinylidene fluoride (PVDF) membrane-transferred proteins are incubated with specific antibodies, and the protein of interest is detected by using a fluorescent agent. Remarkably, some of the modifications of proteins related to RCDs could also be detected through western blots, such as phosphorylation, acetylation, ubiquitin, etc. For example, the phosphorylation of RIPK1 could be analyzed via western blotting with special antibodies for research into the regulation of RIPK1 activation by TAK1-mediated phosphorylation, which modulates apoptosis and necroptosis (Geng et al., 2017).

#### Flow Cytometry

Requiring a high specificity, flow cytometry analysis is utilized to detect the specified cells through an assay of specific markers, volume, form, or any other signal characteristics (Bandura et al., 2009). For detection of apoptosis, PI is excited with a xenon or mercury arc lamp or with the 488 lines of an argon-ion laser and can be detected in the particular fluorescence channel (FL2 or FL3) of a flow fluorocytometer (FACSCalibur flow fluorocytometer, Becton Dickinson) (Bergamaschi et al., 2019). Traditionally, Annexin A5 (A5, PtdSer binding protein) combined with either PI or 7-aminoactinomycin D (7-AAD, membrane impermeable DNA binding dyes) stains are used in flow cytometry to distinguish apoptosis from necrosis (Koopman et al., 1994; Vermes et al., 1995; Broaddus et al., 1996). For the detection of ferroptosis, flow cytometry-based analysis is easy to carry out and is highly sensitive to measure lipid peroxide levels in live cells with the BODIPY^TM^ 581/591 C11 dye (Martinez et al., 2020). This technique is not only used for the separation of positive cells and the capture of special free contents such as vesicles, but also for identifying molecular biomarkers. Recently, flow cytometry assays have been improved to detect and quantify the various forms of RCDs, such as a three-color flow cytometry analysis that has been reported to detect necroptosis and apoptosis in the early and late-stage, and receptor interaction protein 1 (RIP1)-dependent apoptosis simultaneously in a single cell through targeting proteins like caspase-3 and receptor interaction protein 3 (RIP3), and detecting cell viability (Bergamaschi et al., 2019).

#### Enzyme-Linked Immunosorbent Assay (ELISA)

In the case of biomarkers for RCDs, ELISA is a sensitive, cost-effective and practical option for detection and quantitative or qualitative analysis based on the production of monoclonal or polyclonal antigen-specific antibodies and radioimmunoassay techniques. The ELISA technique is often used to detect RCD-related proteins such as caspase-3/7 up-regulation during apoptosis or cell-free DNA and nucleosomes (Roth et al., 2011), but there are still some constraints that should be of concern, such as the effect of temperature and time on sample storage since samples stored at −70°C lead to an annual loss of 7% (Holdenrieder et al., 2010).

#### Cryo-EM

Recently, to present the microenvironment of a protein as much as possible, cryo-EM has been found to be a powerful tool and has been recently used in the research into structural molecular and cellular biology in three-dimensional structures, which was selected by Nature Methods as the Method of the Year 2015, and the Nobel Prize in Chemistry 2017 (Bendory et al., 2020; Garcia-Nafria and Tate, 2020; Guerrero-Ferreira et al., 2020). It provides benefits for a better understanding of how proteins and/or other biological macromolecules are involved in their complex network (Bendory et al., 2020). Cryo-EM is regarded as a suitable method for identifying the structure of isolated biomolecular complexes ranging from a protein sized several tens kilo-Daltons to a virus particle-sized many mega-Daltons and to a whole cell with sub-nanometer resolution (Murata and Wolf, 2018). As for the sample preparations, cryo-EM requires a much smaller amount of sample with tomographic slices of 10-nm thickness; it accepts large image datasets (such as single protein molecules, large protein complexes, thin-protein crystals, virus particles, helical fiber complexes, bacteria, cells, and even entire tissue sections); and near-atomic 3D maps of isolated proteins could be provided (Danev et al., 2019). Kate et al. have shown the reconstructed cryo-tomogram of apoptotic herniating mitochondria to research the classical intrinsic apoptosis pathway by using cryo-EM (McArthur et al., 2018). Meanwhile, cryo-EM has also been used to reveal the morphology of the pores and determine the localization of Bax labeled with nanogold, which allows for understanding the mechanisms of pore formation induced by Bax in apoptosis, necroptosis or ferroptosis (Kuwana, 2019). The proteins of RCD, including their connected macromolecules, microenvironment and even the related signal pathways, can be visualized by using cryo-EM. For example, the filament structure of caspase-8 tandem death effector domain was determined through cryo-EM to achieve a presentation of extensive assembly interfaces and to further confirm with structure-based mutagenesis its filament formation *in vitro*, as well as Fas-induced apoptosis and ASC-mediated caspase-8 recruitment in cells (Fu et al., 2016).

Also, researchers adapted some targeted approaches for the special marker assays, such as the terminal -deoxynucleotidyl transferase mediated nick end labeling (TUNEL) assay that is designed to detect DNA degradation in the late stages of apoptosis. Recently, circulating molecules (e.g., non-coding RNAs, released proteins, heteromeric complexes, enzymatic activity, subcellular vesicles, *etc*.) released from special tissues were found to be detectable in the cerebrospinal fluid, plasma, serum, and any other body fluids, which suggests noninvasive clinical applications.

### Biomarkers for Detecting RCDs

#### Cell Surface Markers

##### PS (PtdSer) exposure vs. membrane permeability

PS (PtdSer), as a vital content in eukaryotic membranes, is the major anionic phospholipid accounting for 2–10%. PtdSer is generally not externally exposed in normal cells and the exposure of PtdSer could be a hallmark for stressed and dying cells, and a key signal for the removal of apoptotic cells through neighboring phagocytic cells (e.g., macrophages or neutrophils). That is, PtdSer exposure not only occurs in apoptosis but also in other types of cell death, such as necroptosis and autophagy (Galluzzi et al., 2012). It is an interconnected process between the exposing PtdSer and the loss of plasma membrane integrity, whose detections are discussed above. As for PtdSer, Annexin V, one of the imaging probes targeting DNA, is widely used to detect apoptosis when it comes to radionuclide imaging. It is worth mentioning that Annexin V, a nonglycosylated membrane protein probe, has been used as one of the few cell death imaging agents reaching phase II/III clinical trials (Nguyen et al., 2012). Synaptotagmin-I (SynI), synaptic vesicle-related protein, provides another point for probe design by binding to the negatively charged phospholipids PS and phosphatidylinositol in the presence of Ca^2+^ ions (Alam et al., 2010).

##### Pannexin 1 (PANX1)

The pannexin (Panx) family, consisting of 3 members (Panx1 as the most extensively studied one, Panx2 and Panx3), has been reported as playing a vital role in extracellular ATP release (Baranova et al., 2004). In apoptosis, Panx1 being activated through cleavage mediated by caspase at the C terminus releases ATP as a “find me” signal, which is necessary for macrophage recruitment (Chekeni et al., 2010). Meanwhile, the activation of Panx1 channels contributes to the increase in plasma membrane permeability and the formation of Ca^2+^-permeable pores at the endoplasmic reticulum, leading to Ca^2+^ leakage and favoring mitochondrial Ca^2+^ uptake, which conveys cytochrome c to the cytosol to induce apoptosis (Chekeni et al., 2010). During research, Panx1 inhibitor carbenoxolone (CBX), pharmacological inhibition or small interfering RNA are used for indirectly inspecting the results *in vivo* and in *vitro*, suggesting PANX1 as a plasma membrane channel that could mediate the regulated release of find-me signals and selective plasma membrane permeability during apoptosis (Chekeni et al., 2010). The quantitative analysis of Panx1 could be detected through qPCR as mRNA levels for *PANX1*, and IHC or western blot for protein expression, whose function is analyzed using patch-clamp methods (Huang et al., 2020). In pyroptosis, caspases-3 and-7 and caspase-11 could not only contribute to cleaving the CT moiety of Panx1, resulting in channel opening and extracellular ATP release, but also induce K+ efflux and the activation of NLRP3 inflammasomes to process and subsequently release IL1β (Yang D. et al., 2015). Dahai et al. identified that Pannexin-1 worked critically for ATP-induced pyroptosis and was induced *via* cytosolic LPS using immunoblotting analysis with or without CBX, probenecid and trovafloxacin (one of the inhibitors of pannexin-1) (Yang D. et al., 2015). As for autophagy, extracellular ATP and engulfing dying cells in the Panx1-dependent pathway could not only recruit immune cells, but was also involved in the pathway of the inflammasome activation in macrophages in which a short hairpin (sh)RNA method for silencing pannexin-1 channels during co-incubation of macrophages with dying autophagic cells leads to the inhibition of ATP release and inflammasome activation (Ayna et al., 2012). By means of inhibitor targeting the key molecule involved in the pathway of RCDs, this reverse validation is also widely accepted, such as CBX or small interfering RNA being used to inhibit Panx1 for apoptosis detection through qPCR and ELISA.

#### Intracellular Markers

##### Oligonucleosomal DNA fragmentation

DNA fragmentation to 180-200 bp and a weight more than 50 kbp is considered as a feature that clearly distinguishes apoptosis (Walker et al., 1999). There are various biochemical techniques to detect DNA ladders, including general-use agarose gel electrophoresis or flow fluorocytometric, which benefits from not being time-consuming and allowing for individual cell analysis. According to different technical principles, there are three main routine assays that were developed to detect DNA fragmentation: DNA ladder assay, TUNEL assay, and comet assay. Firstly, the DNA ladder assay: the DNA fragments (180–200 bp) could be separated into the “DNA ladder” pattern on agarose gel electrophoresis. The DNA ladder assay is used in conjunction with commercial kits that lead to a faster, more accurate, and sensitive assay, but at a greater cost (Micoud et al., 2001). Another approach is the TUNEL assay, which is utilized as a standard histochemical method for tissues, adherent cell lines, and suspension cell lines (Cuello-Carrion and Ciocca, 1999). Different from the DNA ladder assay, the TUNEL assay is more sensitive for the use of specific markers [e.g., bromodeoxyuridine labeling (BrdU), fluorescein labeling, thymidine analogs, 5′-ethynyl2-deoxyuridine labeling (EdU) (Wu et al., 2012), *etc.*] combined with other techniques, such as FM, flow cytometry, or laser scanning cytometry (Darzynkiewicz et al., 2008). As for the comet assay, also named ‘single cell gel electrophoresis assay’ (SCGE), it has been developed in genotoxicity testing with rapid detection of DNA repair or damage in a single cell (Collins, 2004). According to the quantification of the fluorescent signal in the core (formed of macromolecules and unfragmented DNA) and the tail (formed of predominantly single-stranded DNA) as well as the DNA damage, comets are divided into five groups: none or very low damage, low damage, medium damage, long DNA migration, apoptotic or necrotic DNA migration (Konca et al., 2003).

Although optimized approaches have been developed, various limitations are still a challenge. For instance, DNA damage not only appears in the apoptotic process but also exists in necroptosis, leading to false-positive assay results. And if there is no DNA ladder pattern, it cannot be proven that there is no apoptosis occurring in the sample since it can result from an event of the internucleosomal cleavage of DNA occurring in late apoptosis (Cohen et al., 1992; Collins et al., 1995). There are sometimes mushrooming cases where it is absent with internucleosomal DNA degradation during apoptotic or apoptotic-like cell death so that the intensity of DNA fragment labeling in these assays will be inadequate to distinguish apoptosis (Cohen et al., 1992; Catchpoole and Stewart, 1993; Knapp et al., 1999).

##### Caspase activation

Caspases, as one of the cysteine aspartate-specific proteases, play a vital role in the early stages of apoptosis, and are regarded as general biomarkers to identify apoptosis. They are synthesized as zymogen precursors that consist of an amino-terminal domain of variable length followed by a p20 and a p10 unit containing the residues. These amino-terminal regions in initiator caspases obtain a caspase recruitment domain (including CARD; caspases 1, 2, 4, 5, 9, 11) or death effector domains (including DED; caspases 8, 10), which are essential for substrate recognition and catalytic activity. After being activated by proximity-induced auto-proteolysis or cleavage via upstream proteases in an intracellular cascade, an active heterotetramer is formed and induces the following pathway (Lamkanfi et al., 2002; Ramirez and Salvesen, 2018). According to the functions and domain architecture, the caspase-family members are classified into two parts: inflammatory (including caspases-1, -4, -5, and -11) or apoptotic (caspases-3, -6, -7, and caspases-8, -9, -10) (Van Opdenbosch and Lamkanfi, 2019). The detection of caspase activation has been used to identify the mitochondrial death pathway of apoptosis in cell-free systems (Chandra and Tang, 2009). Besides caspases, other proteins in response to apoptotic stimuli are also targeted as an indicator, such as Bid, Fas-associated protein with death domain (FADD), Bcl-2 and Bax, cytochrome c, high-mobility group box 1 (HMGβ1), nuclear factor kappa B (NFκB), poly [ADP-Ribose] polymerase 1 (PARP) and other apoptosis-related proteins (Nicholson et al., 1995; Gyulkhandanyan et al., 2012; Del Re et al., 2019; Yang L.L. et al., 2019; Zahran et al., 2020). In pyroptosis, it has been reported that caspase-1 could mediate the cleavage of the cytosolic protein gasdermin D (GSDMD), resulting in promotion of the formation of GSDMD membrane pores and cell lysis, which sheds much-needed light on the necrotic execution mechanism of pyroptosis (de Vasconcelos et al., 2019). As a caspase-independent pathway, necroptosis is also regulated through the caspase regulators in which caspase 8 could work as a potent inhibitor of necroptosis mediated through a heterotrimeric complex involving caspase 8, Fadd, and the long isoform of caspase 8- and FADD-like apoptosis regulator (Dillon et al., 2012). Caspase activity and these related proteins can be detected through flow fluorocytometry, the cleavage of an *in vivo* caspase substrate, FLIM and light microscopy, the Promega Caspase-Glo 3/7 assay, and ELISA, and meanwhile q-PCR is used at the level of the RNAs of the related proteins during cell death. An example of caspase detection, the Promega Caspase-Glo 3/7 assay (Promega Corp., Madison, WI, United States), is utilized for detection, adopting a proluminescent caspase substrate for caspase detection (Mfotie Njoya et al., 2018).

##### Bid cleavage and the expression of the Bcl-2 protein family

The integrity of the major outer-membrane protein (MOMP) is closely regulated by a group of proteins belonging to the Bcl-2 family and encoded by the *BCL2* genes, which consists of pro-apoptotic and anti-apoptotic members (e.g., Bcl-2 and Bcl-xL) or other classes (e.g., BH3-only proteins with Bcl-2 homology (BH) domains only) (Cheng et al., 2001; Wei et al., 2001; Chipuk et al., 2010). Generally, it is believed that Bcl-2, Bax and Bak are of great importance in the intrinsic pathway of apoptosis and the Bcl-2/Bax ratio or the level of Bcl-2 is considered as a hall marker for apoptotic detection (Musumeci et al., 2015; Saraste and Pulkki, 2000). The extrinsic pathway of apoptosis, also known as the death receptor pathway, is closely related to the intrinsic pathway (mitochondrial pathway), since the truncated Bid (tBid) protein translocates to the outer mitochondrial membrane (OMM) to promote RCD by engaging Bax/Bak, resulting in MOMP and subsequent caspase-9 activation (Wu X. et al., 2020). Similar to second mitochondria-derived activator of caspases (SMAC) mimetics, MOMP could trigger tumor necrosis factor (NF-κB-dependent production) and coincidentally induce an alternative form of cell death, namely necroptosis (Giampazolias et al., 2017). Remarkably, the Bid is from both the cytosol and the organelle fraction. Because the form of Bid induced by anti-Fas antibodies stays in the cytosolic fraction briefly and only in small amounts, most of the Bid has been associated with the organelle fraction (Krysko et al., 2008). Both the Bid cleavage and the expression of the Bcl-2 protein family are able to be detected through western blot, ELISA, and flow cytometry.

##### Cytochrome c release

The release of cytochrome c from the mitochondria is a central signal in the intrinsic pathway of apoptosis mediated through OMM permeabilization. The release of cytochrome c activates caspase-3 or -7 *via* the apoptosome, leading to the formation of cyt-c, caspase-9 and Apaf-1, after which apoptosis is induced (Green and Kroemer, 2005; Kroemer et al., 2007). The detection of cytochrome c serves as a biomarker of apoptosis and is also important to understand certain diseases at the cellular level. To detect the level of cytochrome c, multiple existing techniques are adopted, including western blot, ELISA, high-performance liquid chromatography (HPLC), spectrophotometry and flow cytometry. As a special detection method for cytochrome c, fluorescent aptamer/carbon dot-based assays are regarded as a simple, sensitive, rapid and selective label-free assay for apoptosis detection employing the principle of a connection between the surfaces of Carbon Dots (CDs) and nucleic acid aptamer biomolecules. It is very sensitive and selective for apoptotic detection with a 25.90 nM limit in detection and a linear range of 40-240 nM (Ghayyem and Faridbod, 2018). Notably, cytosolic fractions and the organelles should be separated using a mild detergent, digitonin (concentration of 0.02%), in order to leave the mitochondria and lysosomes intact to prevent cytochrome c release from the mitochondria, resulting in artifacts of organelle preparation (Krysko et al., 2008). Moreover, cyt c could accumulate in the culture supernatant during secondary necrosis of anti-Fas-stimulated cells, and cyt c could also accumulate in the culture supernatant from the moment that the plasma membrane loses its integrity in the late necrotic phase of TNF-stimulated cells (Denecker et al., 2001).

##### The expression and phosphorylation status of RIPK1, RIPK3, and MLKL

Various factors work as key participants in necroptosis, namely in a caspase-independent pathway, such as RIP1, RIP3, and MLKL (Galluzzi et al., 2014; Ding et al., 2015; Wu X. et al., 2020). Combined with traditional methods of detection of necrosis (morphological features, the intracellular-component release, and biochemical features involved in necrosis), specific biomarkers in necrosis allow for more accurate detection of necrosis or even a measure with greater potential for clinical implications (Vanden Berghe et al., 2013; Lu et al., 2017). It has been reported that necrosis is sometimes related to upregulated RIPK1, RIPK3, or MLKL mRNA or protein expression levels *in vivo* in various diseases or physiological conditions (Guo et al., 2020). Activated forms of RIP1, RIP3, and MLKL have emerged as optimal biomarkers for both distinguishing necrosis and the diagnosis or prognostic assessment of diseases related to necrotic injury (He et al., 2016). Some of the specific kinase inhibitors and inducers have been widely used for research into necroptotic signals, such as drug-induced forced dimerization of RIPK1/RIPK3, a means to directly activate RIPK1 or RIPK3 (Rodriguez and Green, 2018). Tissues or cells with increased expression levels of RIPK1, RIPK3, and MLKL may indicate a predisposition to necrosis. However, there are some exceptions to challenge these biomarker indicators while non-necroptotic functions of increased RIPK1 and RIPK3 or necrosis induced without RIPK1 and RIPK3. In detail, the increased expression of RIPK1 and/or RIPK3 could contribute to inflammatory processes as an independent necrotic pathway by regulating pro-inflammatory cytokines (e.g., TNF, IL-1β, and IL-18) (Christofferson et al., 2012; Moriwaki and Chan, 2014; Wong et al., 2014). The phosphorylation status of RIPK3 on S227, and MLKL on Ser358 and Thr357, is considered to reflect activation toward necroptosis (Degterev et al., 2008; McQuade et al., 2013; Wang et al., 2020b). For this biomarker, kinase-dead RIPK3 could function as an anti-necroptotic factor, and similarly, the negative regulation also includes caspase 8, cellular FLICE-inhibitory protein (c-FLIP), chromatin immunoprecipitation (CHIP), MAPK (mitogen-activated protein kinase)-activated protein kinase 2 (MK2), pellino E3 ubiquitin protein ligase 1 (PELI1), and ABIN-1 (A20 binding and inhibitor of NF-κB) (Wang et al., 2018c).

##### The levels of Atg and LC3

For monitoring autophagy *in vivo*, the ‘core’ *Atg* genes knockout mouse, GFP-LC3 transgenic mouse, TfLC3 transgenic mouse, and GFP-LC3-RFP-LC3ΔG transgenic mouse are often used as animal models to detect specific protein markers (Kuma et al., 2017). These markers are experimentally adapted as follows: (1) identification of autophagy-related (ATG) genes and proteins related to the initiation of autophagy could be used to assess the activity of autophagy and to study the role of autophagy in the pathophysiological process; (2) for the LC3 (LC3B) and GABARAP family, the ratio of lipidated LC3 (LC3-II) to free LC3 is usually utilized to reflect the number of autophagosomes forming at any given time; 3) tandem fluorescent-tagged LC3 (tfLC3) reporters are used as single-molecule probes to detect autophagosomes labeled with yellow (mRFP and GFP) and autolysosomes labeled with red (mRFP only). Other detections include the GFP-LC3-RFP-LC3ΔG probe, etc. However, some of the Atg proteins may have autophagy-independent functions and the underlying mechanisms are not elucidated so far, such that the detection of autophagy is still facing challenges (Paunovic et al., 2018; Tan et al., 2019). As for specified autophagy, such as mitophagy, it is related to signaling proteins, such as TOMM20, TIMM23, LC3, and p62 (Wang et al., 2020c).

Meanwhile, the levels of beclin have been used as a marker of autophagy, but there should be more prudent use of it, since in some cases, beclin levels can be transcriptionally upregulated to wild-type levels even in beclin heterozygous or beclin allelic loss cells (Murugan and Amaravadi, 2016). The Atg family are also key proteins, but they are not a reliable reflection of autophagic flux unless combined with LC3 (Murugan and Amaravadi, 2016). According to the functions of LC3 processing for autophagosomes, both its formation and features, western blotting is widely used in autophagy monitoring with antibodies against LC3-I and LC3-II. qPCR-based approaches have also been used for detecting the mRNA levels of autophagy regulators, such as beclin-1, ATG1, DRAM, and LC3 (Kuma et al., 2017).

##### A brief introduction of biomarkers in pyroptosis

Regarding pyroptosis, there are some proteins used as experimental markers, including caspase-1, propidium, IL-1β, GFP, tdRFP, and LDH. Among these, PI staining is always regarded as a proxy for membrane rupture as it is often found simultaneously for these two things, namely the membrane rupture and the activation of the gasdermin pore (Jorgensen et al., 2016). Recently, researchers have evaluated the reliability of the detection as the molecules and proteins that we detected may pass through the membrane rupture, or through the gasdermin pore, or both (Kovacs and Miao, 2017). For instance, PI and IL-1β (4.5 nm; 5 nm) are small enough to pass through the gasdermin pore (10-15 nm), resulting in a situation that detected significant amounts of PI before the membrane rupture happened (Ding et al., 2016; Liu et al., 2016). On the contrary, tdRFP and LDH are larger, so they seem to be released by the membrane rupture event (Shaner et al., 2004; Russo et al., 2016).

##### A brief introduction of biomarkers in ferroptosis

As a non-apoptotic form of RCDs, ferroptosis is characterized by iron-dependent accumulation of toxic lipid peroxides in plasma membranes. The cystathionine-β-synthase (CBS) is a marker of transsulfuration pathway activity involved in ferroptosis (Wang et al., 2019b). The lipid peroxide level is closely related to ferroptosis, so it provides a useful means to detect ferroptosis in biological samples, such as measuring the level of cellular lipid peroxide via flow cytometry assays, immunostaining, or colorimetric assays (Uchida et al., 1993: Yagi, 1998; Drummen et al., 2002). Meanwhile, intracellular concentrations of iron and Fe^2+^ and the mitochondrial membrane potential are also used as indicators to distinguish the induction of ferroptosis (Wang et al., 2019b, 2020a). Furthermore, increased ACSL4 is required for lipotoxicity in ferroptosis, and unlike the other ACSL members, it seems to be a marker of ferroptosis since ACSL4 is remarkably downregulated in ferroptosis-resistant cells (e.g., LNCaP and K562) (Yuan et al., 2016; Wenzel et al., 2017). As for the specific detection of ferroptosis, the changes of the mitochondria are regarded as one of the most important indicators, including the morphological and biochemical changes. Increased intracellular concentrations of iron and Fe^2+^ can be monitored by kits, such as an Iron Assay Kit (Sigma Aldrich). Increased mitochondrial superoxide can be detected using a specific fluorescent probe, such as MitoSOX^TM^ Red Mitochondrial Superoxide Indicator for live-cell imaging (Invitrogen). Decreased mitochondrial membrane potential is measured by some kits, such as the Mitochondrial Membrane Potential Kit MAK-159 (Sigma Aldrich) for monitoring fluorescence intensity levels (λ_*ex*_ = 490/λ_*em*_ = 525 nm) and (λ_*ex*_ = 540/λ_*em*_ = 590 nm) for the ratio analysis (Wang et al., 2019b).

##### A brief introduction of biomarkers in NETosis

Based on the specific molecules involved in the pathway during NETosis, co-localization of neutrophil-derived proteins (such as myeloperoxidase (MPO) and proteinase 3 (PR3)), and extracellular DNA would suggest the presence of NETosis (Kessenbrock et al., 2009; Nakazawa et al., 2012). Additionally, citrullinated histones could be regarded as a marker for indicating NETs formation. Histones are citrullinated by PAD4 which transport from the cytoplasm to the nucleus activated *via* ROS generation and calcium influx (Remijsen et al., 2011; Wang and Wang, 2013). And cfDNA could also function as one form of NETs remnants which could be detected using PicoGreen^®^ (Zhang et al., 2014).

#### Release of Extracellular Markers Into the Supernatant or Circulating Biomarkers

The biomarkers released from the cells could remain in the tissue fluid or enter bodily fluids as circulating markers. Some of them could be detected *in vitro* from the supernatant of the samples such as from primary or secondary necrotic cells. Similarly, the monitoring of biomarkers *in vivo* in a liquid biopsy (e.g., plasma samples) also provides another method for detection. These kinds of biomarkers include caspases-3 and -7, high mobility group box 1 protein (HMGB-1), and CK18. Identifying the RCDs *via* the circulating biomarkers is a valid noninvasive alternative in clinical applications.

##### Caspase-3

Caspase-3, a cysteine protease, could retain its tetrapeptide sequence DEVD (a distinct amino acid sequence of Asp-Glu-Val-Asp), which provides cleaving activity for a long time in various extracellular fluids (Hentze et al., 2001). The p20 subunits of caspase-3 and -7 have been detected in the culture medium following secondary necrosis, whereas there are no such markers but only procaspase-3 and -7 in necrosis (Denecker et al., 2001). Thus, it has been proposed as a specific releasing and circulating biomarker to indicate apoptosis in the tissues (Denecker et al., 2001). Apoptotic cells in the tissue could be measured through various technologies, such as the TdT-mediated X-dUTP nick end labeling method and IHC using antibodies against active caspase 3 or caspase-cleaved proteins to identify the degree of apoptosis (Deng et al., 2019; Kunac et al., 2019).

##### Cytokeratin 18

CK18 releases a protein cleaved via the effector caspases at two distinct sites (Asp238 and Asp396) during cell death (Ku et al., 2016). The method named the M30-Apoptosense assay (Peviva AB, Bromma, Sweden) is used to measure the caspase-cleavage CK18 at Asp396 (CK18Asp396-NE M30 neo-epitope) and to specifically discriminate between apoptotic and necrotic cell death (Cummings et al., 2008). The circulating ccCK18 was quantitated through a specific ELISA combined with the antibody, which showed a positive signal in M30 and M65 to indicate apoptosis, whereas an exclusive positive signal in M65 was used to indicate necrosis (Kramer et al., 2004). The time and temperature requirements of this test may bias the results since it requires the samples be placed immediately on ice in order to avoid artificial cell death between acquisition and processing (Greystoke et al., 2008).

##### HMGB-1

High-mobility group box 1, an architectural chromatin-binding factor, could bind to DNA for increasing protein assembly targeted to specific DNA. It is a protein secreted through activated monocytes or macrophages and is passively released *via* necrotic or damaged cells, but it should be mentioned that HMGB-1 cannot be found in apoptotic cells even after undergoing secondary necrosis and partial autolysis, resulting in a failure to promote inflammation even if not cleared promptly by phagocytic cells (Scaffidi et al., 2002). HMGB-1 released from necrotic cells could activate the macrophages *via* the toll-like receptor 2 (TLR2) and toll-like receptor 4 (TLR4) pathway (Park et al., 2004). HMGB-1 can also chemoattract and activate dendritic cells (DCs) with processing and presentation of tumor antigens (Apetoh et al., 2007; Yang et al., 2007).

Other markers released from cells may also provide indicators for RCD detection, such as DNA laddering signals. The “DNA laddering” resulting from fragmented DNA is cleaved through DNase activated *via* the inhibitor of caspase-activated DNase (iCAT) (Krysko et al., 2008). The 166 base pairs (bp)-length cell-free DNA (cfDNA) consists mainly of nucleosome-protected DNA being released from apoptotic tumor cells into the bloodstream, which might be used in the detection of RCD, providing a target for clinical applications (Krysko et al., 2008; Ulz et al., 2016).

#### Potential Markers

Recently, non-coding RNA (ncRNA) has been confirmed to have a strong correlation with RCDs, and a positive correlation or a negative correlation might be developed as a useful detection method for monitoring RCDs. For instance, miRNAs (microRNA, one of the non-coding RNAs, 21-23 nucleotides long) is closely related to RCDs, like miR-21 is related to necrosis, miR-137 is related to ferroptosis (Park et al., 2004), miR-184 is related to apoptosis, miRNA-335-5p is related to autophagy, and miR-223 is related to pyroptosis (Wang et al., 2015; Afonso et al., 2018; Luo et al., 2018; Zhang Y. et al., 2018; Zhong et al., 2019). Other ncRNAs like long-coding RNA (lincRNA) and circulating RND (circRNA) may also present the same effect (Zhou et al., 2019; Tao et al., 2020). However, some ncRNAs in RCDs of different healthy or pathological cells or tissues emerge with different expression patterns, so there is still controversy about these biomarkers functioning as indicators to detect RCDs. It is also worth noting that RCDs such as apoptotic cells could release vesicles as apoptotic microvesicles and exosomes-like vesicles that are smaller than ApoBDs (apoptotic bodies). Extracellular vesicles (EVs) like exosomes, microvesicles (MV), ApoBDs (1–5 μm in diameter) and ApoMVs (<1 μm in diameter) released from special cells or tissues may have a close connection with RCDs, which suggests an occurrence of RCDs in pathophysiological processes (Caruso and Poon, 2018; Pavlyukov et al., 2018; Grant et al., 2019; Nooshabadi et al., 2020; Qin et al., 2020). Marat S. et al. investigated the extracellular vesicles secreted by apoptotic glioblastoma cells (apoEVs) for apoptotic research associated with a phenotypic shift of the recipient surviving tumor cells (Pavlyukov et al., 2018).

Notably, cell death has been closely linked to inflammation through various signals such as RIPK1, RIPK3, FADD, FLIP and caspase 8. These molecules are incorporated into compatible and exceedingly dynamic Toll-like receptor, retinoic acid-inducible gene I (RIG-I)-like receptors, and NOD-like receptor which have roles in switching from inflammation to cell death, or perform a programmed execution of both. For example, the overexpression of caspase 11 could induce apoptosis (Wang et al., 1996), and it is also linked to inflammation as an upstream regulator of caspase 1 to promote both pyroptosis and pro-IL-1β processing, or as a molecule releasing after lipopolysaccharide (LPS) or tissue injury (Kang et al., 2000). Caspase 8, involved in the extrinsic pathway of apoptosis, could lead to the development of systemic inflammation (Wallach et al., 2014). Pyroptosis is initiated in response to inflammasome activation in the mobilization of the canonical and/or non-canonical pathways (Bergsbaken et al., 2009). The activation of RIPK3 is crucial for necroptosis induced by TNF. The role for RIPK1 is closely related to other molecules as TNFR1, FADD and caspase 8, and TRIF, IFN and RIPK3 related necroptosis to inflammation, or other types of cell death (Dillon et al., 2014; Kaiser et al., 2014; Rickard et al., 2014). As for autophagy, its proteins play a vital role in inflammation, in which the LC3 conjugation system (vital molecules involved in autophagy, i.e., ATG3, ATG5, ATG7, and ATG16L1) is related to the activation of cells with IFN-γ during inflammation (Zhao et al., 2008; Li et al., 2021). LAP could induce pro-inflammatory gene expression and trigger STING-mediated type I interferon responses in tumor-associated macrophages (Cunha et al., 2018). Compelling evidence showed that ferroptosis have an effect on inflammation where ferroptosis inhibitors exerted anti-inflammatory effects in certain diseases (Sun et al., 2020). Besides the events discussed above, there is still a lot of evidence showing a close connection between cell death and inflammation which suggest these specific inflammation-related factors could function as indicators for adjunctive detection of cell death.

All in all, biomarkers provide pathway-specific measures to distinguish different types of RCDs, but there are still some issues: (1) Protein specificity: the overlap or connected markers involved in different types of RCDs; (2) Misleading results: the specific biomarkers are related to other responses not involving the expected cell death, and the occurrence of cell death is not connected with the targeted proteins; (3) Simultaneous events, such as the induction of cell death may result in another cell death or simultaneous events of two or more types of cell death; (4) Limited detection technology: some of the subtle changes require more sensitive and comprehensive detection techniques or combined manipulations of pathway-specific markers in cell death pathways.

## Monitoring and Measuring RCDs From *in vivo* to Clinical Applications

### Monitoring and Measuring RCDs *in vivo*

It is hard to mimic the real microenvironment of cells *in vitro* entirely, and the detection of RCDs *in vivo* is vital for research. The gamma camera imaging, CT, MRI/MRS, PET, and radionuclide imaging methods are usually used to monitor the biochemical and physiological processes of RCDs *in vivo* (Brauer, 2003; Huang et al., 2017). As a common example, MRI is used to monitor the volume reduction of a tumor induced by RCDs. For example, ^23^Na MRI might be a sufficiently useful and practical method in clinical or laboratory detection of apoptosis. ^23^Na MRI may be sensitive to high concentrations of Na^+^ in tissues and have a close connection to electrolyte-macromolecular interactions during RCDs (Brauer, 2003). The monitoring of cell death *in vivo* provides evidence as to whether or not and how much damage occurs and then guide the clinical assessment and treatment based on the severity of the diseases and the drug choice of anti-bacterial or anti-viral agents.

According to various structural and functional perspectives involved in different types of RCD both *in vivo* and *in vitro*, multiple techniques are required for detection with high repeatability, specificity, and precision. The assays related to morphological, biochemical, and functional changes are presented in Supplementary Table 1.

### RCDs Detection in Clinic Usage

Regulated cell deaths are closely related to a variety of diseases, and mostly detection of RCDs has been used for early diagnosis and prognosis assessment through monitoring the quality and quantity of the biomarkers involved in RCDs and the various molecular players also used as drug targets for treatment (Thygesen et al., 2007). During development, both the nervous system and the immunogenic cells of the hematopoietic system particularly rely on the overproduction of the cells; in other words, RCDs play a vital role in the nervous and the hematopoietic system. A harmonious process that integrates proliferation, differentiation, and RCDs maintains normal development and the generation of functional circuitry within the nervous system, with elimination of neurons migrating and innervating improper targets or ectopic areas, and the limiting amounts of pro-survival factors produced by targets (including glia) resulting in optimal target innervation through competition with neurons (Fricker et al., 2018). Once the balance breaks, it may lead to various neurodegenerative diseases with a fundamental pathological feature of cell death, such as strokes with a high number of neurons dying by necrosis (Tian et al., 2019; Zhou et al., 2020). Some researchers have used multiple detection methods to achieve signaling pathway mining and clinical guidelines, such as the detection of RIP3 and phosphorylated RIP3s using western blots, and verifying the necroptosis of retinal ganglion cells using EM in retinal diseases research (Liao et al., 2017).

In the immune system, many potentially dangerous or useless immune cells could be eliminated through RCD pathways such as the positive selection and negative selection of T cells (Opferman, 2008; Anuradha et al., 2013). The functions of the immune system are also connected to RCDs, such as apoptotic cells being efficiently cleared in a quiescent manner through the immune system (Kolb et al., 2017). Similarly, the breakage of the steady state is closely related to various immune diseases, such as apoptosis-related autoimmune disease and autophagy-related immune renal disease (Mihaljevic et al., 2018; Ye et al., 2019). Elsewhere, in the circulatory system, platelet apoptosis is characterized by platelet-derived microparticle (MP) formation and cell shrinkage in different cellular compartments (mitochondria, cytosol, and plasma membrane) or at the whole-cell level, has a strong correlation with platelet-related diseases such as vascular restenosis, atherosclerosis, wound healing, angiogenesis, inflammation and immune responses (Smyth et al., 2009; Semple et al., 2011; Gyulkhandanyan et al., 2012). In many cancer types, the value of the apoptotic index (AI) in diagnosis or prognosis has been developed but the results of its accurate evaluation and/or standard settings are controversial (Becker et al., 2014; Fu et al., 2014). It has been reported that the subsequent development of diseases is tied closely to AI, which may be a useful biomarker for prospective studies (Braga et al., 2016), but the results still remain controversial.

Furthermore, we have summarized the biomarkers of RCDs used in diagnostic and prognostic assessment in Table 4 as well as some related clinical applications in Supplementary Table 2. The detection of biomarkers in RCDs are widely used in these assessments, but there are still challenges for their accuracy and effectiveness of detection, tissue and species specificity, etc. Furthermore, targeting RCDs is beneficial for not only diagnosis and prognostication, but also treatment through using drugs to regulate the proteins or genes involved in the RCD pathway.

**TABLE 4:** Biomarkers of RCDs used in diagnostic, prognostic and research histopathology.

Items	Type	Markers	References
Apoptosis	Diagnostic	Bcl-2, p53, FasL, TNF-α, DNA fragmentation, BBC3, PMAIP1, M30, XIAP, Survivin	Karamitopoulou et al., 2007; Farnebo et al., 2011; Omori et al., 2011; Sen et al., 2015; Heng et al., 2016; Bani-Ahmad et al., 2018; Chen et al., 2019; Henrich et al., 2019; Moledina et al., 2019; Schiffmann et al., 2019
	Prognostic	Bcl-2, Bax, p53, Fas, FasL, caspase-2, caspase-3, caspase-7, caspase-8, caspase-9, TNF-α, DNA fragmentation, Bak, Bok, Bim, PUMA, PMAIP1, MCL1, BCL2L10, TRAIL, TRAIL-R1/2/3, c-FLIP, IAPs	Wang P. et al., 2017; Feng et al., 2018; Huang K.H. et al., 2018
Necroptosis	Diagnostic	NLRP3, AUNIP, RIPK1/3	He et al., 2016; Lee et al., 2016; Yang Z. et al., 2019
	Prognostic	NLRP3, MLKL, RIPK1/3, TLR3/TICAM1, AURKA	Yuan et al., 2015; Yao et al., 2017; Conev et al., 2019; Soleymani Fard et al., 2019; Sun et al., 2019; Malhotra et al., 2020
Autophagy	Diagnostic	LC3, Na+/K+-ATPase, mTOR	Mijatovic et al., 2012; Mete et al., 2018; Sui et al., 2018
	Prognostic	LC3, ATG, BECN1, Na^+^/K^+^-ATPase, AMPK, mTOR	Cao et al., 2016; Cheng et al., 2016; Schläfli et al., 2016; Li et al., 2017; Gajate et al., 2018; Guo et al., 2019
Ferroptosis	Diagnostic	GPX4, ALOX15, SLC7A11, BAP1, HSP90, HSPB1, FANCD2, TP53	Kamal et al., 2004; Guerriero et al., 2015; Saif et al., 2016; Hui et al., 2017; Davidson et al., 2018; Xu et al., 2019
	Prognostic	GPX4, ACSL4, SLC7A11, TFRC, GLS2, DPP4, NCOA4, BAP1, PEBP1, CARS, VDAC2, HSP90, HSPB1, ITGA6, ITGB4, OTUB1, TP53, HSPA5, FANCD2	Wada et al., 2006; Xu et al., 2010; Zhou et al., 2014; Yang Z. et al., 2015; Chen et al., 2016; Luchini et al., 2016; Lu et al., 2017; Sotgia et al., 2017; Sun and Xu, 2017; Zhao et al., 2017; Dimas et al., 2018; Huang Z.C. et al., 2018; Jiao et al., 2018; Kinowaki et al., 2018; Zhang L. et al., 2018; Li M. et al., 2019; Saha et al., 2019; Dinarvand et al., 2020; Feng et al., 2020; Wu G. et al., 2020
Pyroptosis	Diagnostic	GSDMD, caspase-4	Terlizzi et al., 2018; Xu et al., 2018
	Prognostic	GSDMD, LPS, caspase-1, caspase-4, PKA, IL-1β, IL-18	Del Gobbo et al., 2016; Wȩdrychowicz et al., 2018; Gao et al., 2018; Hosonaga et al., 2018; Terlizzi et al., 2018; García de Guadiana-Romualdo et al., 2019; Gil and Kim, 2019

## Conclusion and Perspective

Regulated cell deaths, a controlled cellular process during development, contribute to a balance in physiological conditions for cell clearance, tissue integrity, and homeostasis in multi-cellular organisms, whereas the dysregulation of RCDs results in various pathological conditions, such as neurodegenerative diseases, developmental and immunological disorders, and cancer. RCDs include many types of cell death: apoptosis, necrosis, autophagy, ferroptosis, and pyroptosis, each of which plays an important role in different physiological and pathological conditions *via* various proteins, genes, and cofactors, building a complex cell signaling network. In the last few years, tremendous progress has been made in digging out the secrets of RCD and striving toward translational medicine and precision medicine. Our team has been engaged in this field of RCD for many years (Huang et al., 2013; Shang et al., 2014, 2017; Ding et al., 2015; Adams et al., 2016; Xiong et al., 2016; Li et al., 2016; Liao et al., 2017; Wang et al., 2018b, c, 2020b; Guo et al., 2020; Wu X. et al., 2020). Remarkably, cryo-EM, emerging as a powerful technique, has been used in a growing number of structural determinations for the assays of high-resolution protein structures besides the proteins involved in RCDs (Cheng et al., 2015). Even realistic presentation of the proteins in their native cellular microenvironment has been achieved through cryo-electron tomography (cryo-ET) (Danev et al., 2019; Kuwana, 2019; Wang et al., 2019a). Also, flow cytometry is regarded as a preferred method since it is sensitive, fast and multifaceted, and this method is constantly improving and being integrated into clinical applications, such as image-based flow cytometry (Kranich et al., 2020). So many methods also show great potential in research and clinical applications, such as real-time fluorometry, or PET and single-photon emission computed tomography (SPECT) with specific tracers, multicolor labeling and sophisticated morphometric analysis, etc. (Grootjans et al., 2016; Wimmer et al., 2020). Remarkably, combining the special markers as indicators for RCDs monitoring would lead to more accurate ways to identify various types of RCDs. All in all, these powerful and specific methods will provide us with stronger evidence to describe the pattern of cell death by identifying molecular players and unraveling the biochemical pathways of death.

## Author Contributions

X-MH was the major contributor in reviewing the literature, writing the manuscript, and creating descriptive figures. Z-XL was a major contributor to editing the tables and figures. R-HL and J-QS assisted in literature reviews. KX and QZ was a major contributor in editing the manuscript. All authors read and approved the final manuscript.

## Conflict of Interest

The authors declare that the research was conducted in the absence of any commercial or financial relationships that could be construed as a potential conflict of interest.
