# Supplementary material for: Guidelines for Regulated Cell Death Assays: A Systematic Summary, A Categorical Comparison, A Prospective
Source: Front Cell Dev Biol. 2021 Mar 4;9:634690. doi: 10.3389/fcell.2021.634690 (PMC7970050; doi:10.3389/fcell.2021.634690)
Supplement: Supplementary file 1 [file Table_1.docx]

**Table S1** Methods for the detection of apoptosis based on different structural and functional considerations.

| **Structural and functional consideration** | **Method** | |
| --- | --- | --- |
| **Cytotoxic detection of membrane alterations in apoptotic cells** | Radioactive and non-radioactive assays measuring plasma membrane permeability | Measurement of DNA synthesis [3H]-thymidine  and BrdU incorporation |
|  | Colorimetric assays | Annexin V viability test |
|  |  | Lactate dehydrogenase (LDH) assay |
|  |  | Permeable and impermeable dyes |
|  | Morphological distinction between apoptosis and necrosis | |
| **DNA fragmentation** | In situ end labeling (ISEL) | |
|  | TdT-mediated dUTP Nick-End Labeling | |
|  | DNA laddering | |
|  | Immunological detection of low molecular weight DNA | |
| **Mitochondrial damage** | MTT assay | |
|  | XTT assay | |
|  | Iron Assay Kit | |
|  | MitoSOX™ Red Mitochondrial Superoxide Indicator Fluorescent Probe | |
| **Mitochondrial membrane potential** | ATP energy production | |
|  | Mitochondrial Membrane Potential Kit (MAK-159) | |
| **Cytochrome c release** | Western blotting | |
|  | Cytochrome c enzyme-linked immunosorbent (ELISA) assay | |
|  | Fluorescent aptamer/carbon dots based assay | |
| **Flow and laser scanning cytometry** | Fluorescence activated cell sorting (FACS) | |
|  | Detection of apoptosis related proteins | |
|  | Comet assay | |
| **Mechanism based assays** | p53 protein analysis | |
|  | Replicative DNA synthesis | |
|  | Repair DNA synthesis | |
| **Improvements in the detection of RCDs** | SEM/STEM/TED imaging assay | |
|  | Three colour flow cytometry assay | |
|  | Time-lapse microscopic assay | |
|  | Light scattering flow cytometry assay | |
|  | Automated imaging of cell surface area | |
|  | Loss of cell adherence | |
|  | Novel biomarkers | ncRNA (non-coding RNA) |
|  |  | Released vesicles |
|  |  | Cell-free DNA (cfDNA) |
|  | Chromatin morphology | Intermediates of chromosome condensation |
|  |  | Genotoxic chromatin changes |
