# Supplementary material for: Guidelines for Regulated Cell Death Assays: A Systematic Summary, A Categorical Comparison, A Prospective
Source: Front Cell Dev Biol. 2021 Mar 4;9:634690. doi: 10.3389/fcell.2021.634690 (PMC7970050; doi:10.3389/fcell.2021.634690)
Supplement: Supplementary file 2 [file Table_2.docx]

**Table S2** Biomarkers of RCDs used in diagnostic, prognostic and research histopathology.

| **Marker** | Type | Implication | Refs |
| --- | --- | --- | --- |
| **Apoptotic index (AI)** | Diagnostic | AI shows its potential as a quantitative method to make the diagnosis of GVHD. | [1-4] |
|  |  | AI was investigated as predictor of gastric carcinoma, cervical cancer and breast cancer. | [5-11] |
|  | Prognostic | AI could be used as potential biomarker for prognosis of osteosarcoma. | [12, 13] |
|  |  | The subsequent development of GTN is tied closely to the AI, which may be an useful biomarker for future prospective studies. | [14, 15] |
|  |  | Previous data on the prognostic significance of AI in NSCLC are limited and largely controversial. | [16, 17] |
| **Bcl-2** | Diagnostic | Bcl-2 could be used for the differentiation of FL and follicular hyperplasia. | [18-21] |
|  |  | Bcl-2 is a valuable biomarker to detect immature enteric ganglion cells in PIPO. | [22, 23] |
|  | Prognostic | Bcl-2 overexpression in diseases represents a common survival adaption in cancer. | [24-27] |
|  |  | Bcl-2 has its prognostic utility in DLBCL. | [28-30] |
|  |  | Bcl-2 expression is a useful prognostic marker of ameloblastomas. | [31] |
| **Bax** | Prognostic | High Bax expression is a good prognosticator for patients of colorectal cancer. | [32] |
|  |  | The ratio of Bcl-2/Bax could be used as an effective biomarker to predict the prognosis of OSCC. | [33] |
|  |  | Evaluation of Bax and Bcl-2 expressions and their co-expression could be developed as a prognostic indicator for cervical cancer. | [34] |
| **Bcl-6** | Diagnostic | Bcl-6 overexpression is frequently observed in FL, DLBCL, Burkitt’s lymphoma. | [35-37] |
|  |  | CD10 and BCL-6 immunostaining have an important role in differential diagnosis of FL from RLH and other low-grade B-cell lymphomas. | [38, 39] |
|  |  | Bcl-6 can be used for diagnosing and distinguishing NLPHL from cHL since Bcl-6 is often unexpressed in HRS cells. | [40, 41] |
|  | Prognostic | Bcl-6 protein expression has been associated with a favorable prognosis in DLBCL. | [42-44] |
| **p53** | Diagnostic | p53 has been primarily used to discriminate between neoplastic and non-neoplastic proliferation. | [45, 46] |
|  |  | A protein that inhibits the activity of p53named MDM2 has also yielded diagnostic utility. | [46, 47] |
|  |  | Cytomorphology combined with p53/CK20 immunostaining is useful for the detection of HGUC and LGUC. | [48] |
|  |  | p53 could be used for distinguish NF from DM. | [49] |
|  |  | p53 has also yielded diagnostic value in the distinction of uterine conventional and mLMS from IMT. | [50] |
|  | Prognostic | p53 is a prognostic factor in VSCC and associated with a poor prognosis in endometrial cancer. | [51-53] |
| **Fas** | Prognostic | Fas expression is a poor prognostic factor for breast carcinoma, CRC, RB and urothelial carcinoma. | [54-57] |
|  |  | Fas expression is a favorable prognosticator in primary nodal DLBCL, SPTCL and esophageal cancer. | [58-61] |
| **FasL** | Diagnostic | FasL expression in HRS cells and the absence of FasL in the FDC cluster represent a disturbed microenvironment in the pathogenesis of HL. | [62, 63] |
|  | Prognostic | FasL expression is an independent prognostic marker in OSCC. | [63] |
| **Caspase-3** | Prognostic | The expression levels of Bcl-2, active-caspase-3, and CD95 influence the prognosis of ALL. | [64] |
|  |  | Caspase-3 is a prognostic marker in gastric cancer after curative surgery. | [65] |
|  |  | IL-1β and Caspase-3 expression serve as independent prognostic markers for metastasis and survival in OSCC. | [66] |
|  |  | CC3 expression is a potential prognostic factor for endometrial cancer with PPC. | [67] |
|  |  | Cleaved caspase-3 and/or caspase-3 might be prognostic biomarkers for certain stages of BMSCC. | [65] |
| **Caspase-7** | Prognostic | The loss of Caspase-7 expression is a prognostic marker in the aggressiveness of ccRCC. | [68] |
| **Caspase-8** | Prognostic | Caspase-8 and Caspase-3 expressions in tumor tissues are prognostic markers for CRC patients. | [69] |
|  |  | Caspase-8 and Caspase-9 is associated with poor prognosis in stage II/III colon carcinoma. | [70] |
|  |  | The presence of the CASP8 -652 6N InsDel or the CASP8 Asp302His variant is an unfavorable prognostic factor in colorectal cancer or neuroblastoma. | [71] |
| **PARP** | Prognostic | PARP-1 expression is a prognostic factor in Desmoid-type fibromatosis. | [72] |
|  |  | PARP1 is an independent prognostic factor in BRCA-proficient ovarian high-grade serous carcinoma. | [73] |
|  |  | PARP-1 overexpression is an independent prognostic factor in Adult Non-M3 AML. | [74] |
|  |  | Overexpression of PARP is an independent prognostic marker for poor survival in Middle Eastern breast cancer. | [75] |
|  |  | PTEN, androgen receptors, PARP and tumour promoter (GST-pi, RASSF1, PITX2) methylation have utility in outcome assessment as prognostic indicators. | [76] |
|  |  | BRCA1 and PARP expression have prognostic value in EOC. | [77] |
| **Annexin V** | Prognostic | Twenty-four-hour urinary Annexin V excretion may be a prognostic marker in children with NS. | [78] |
|  |  | 99mTc-HYNIC Annexin-V has prognostic value in SCC of the head and neck. | [79] |
|  |  | ANXA2, A5, A7 and A10 may be potential prognostic biomarkers of liver cancer. | [80] |
| **Gasdermin D(GSDMD)** | Diagnostic | GSDMD plays a key role in the pathogenesis of NASH. | [81] |
|  | Prognostic | Downregulation of GSDMD predicts a good prognosis in NSCLC. | [82] |
|  |  | High expression of CD147 as an unfavorable prognostic marker contributes to tumor proliferation in BC via GSDMD. | [83] |
| **CK18-M30** | Diagnostic | Serum M30 and M65 in patients have diagnostic value with nasopharyngeal carcinoma. | [84] |
|  |  | CK-18, FGF-21, and related biomarker panel have diagnostic value in NAFLD. | [85] |
|  | Prognostic | M30/M65 has prognostic value for outcome of HBV-related ACLF. | [86] |
|  |  | Serum M30 levels are associated with survival in advanced gastric carcinoma patients. | [87] |
| **IAPs** | Diagnostic | XIAP expression in EAC might directly impact on clinical diagnosis and treatment of EAC. | [88] |
|  |  | Survivin showed the most promising biomarker of radioresponse in case of HNC cell lines. | [89] |
|  | Prognostic | Survivin plays a role as a biomarker and potential prognostic factor for breast cancer. | [90] |
|  |  | BIRC6 is a predictor of prognosis in prostate cancer. | [91] |
|  |  | Expression of the IAP protein family acts cooperatively to predict prognosis in human bladder cancer patients. | [92] |
| **TNF-α** | Diagnostic | Inclusion of urinary TNF-α and IL-9 improves discrimination over clinicians' prebiopsy diagnosis and currently available tests for AIN diagnosis. | [93] |
|  | Prognostic | TNF-α receptor 1 expression predicts poor prognosis of DLBCL. | [94] |
| **TRAIL** | Prognostic | Heterogeneous intracellular TRAIL-receptor distribution predicts poor outcome in breast cancer patients. | [95] |
|  |  | Cytoplasmic TRAIL-R1 is a positive prognostic marker in PDAC. | [96] |
| **TRAF6** | Prognostic | TRAF6 promotes the invasion and metastasis and predicts a poor prognosis in gastric cancer. | [97] |
|  |  | Elevated TRAF6 expression levels may indicate a poor prognosis in patients with breast cancer. | [98] |
| **PD-L1** | Prognostic | PD-L1 expression is a promising biomarker for the prognosis of breast cancer. | [99] |
|  |  | PD-L1 expression was an independent predictor for poor prognosis of patients with small cell lung cancer. | [100] |
| **c-FLIP** | Prognostic | c-FLIP could be used as an important tumor marker for personalized cancer therapy and prognostic prediction in invasive breast cancer. | [101] |
|  |  | c-FLIP expression in colorectal carcinomas conveys independent prognostic information in the presence of classical prognosticators. | [102] |
| **GPX4** | Diagnostic and prognostic | GPX4 and GPX7 are possible markers for improving HCC diagnosis/prognosis. | [103] |
|  | Prognostic | The overexpression of GPX4 is an independent prognostic predictor in DLBCL. | [104] |
|  |  | GPX4 might be a new prognostic factor in glioma and be closely correlated with glioma cell proliferation, migration and apoptosis. | [105] |
| **PDCD6** | Prognostic | The PDCD6 gene is a significant prognostic biomarker for advanced gastric cancer patients. | [106] |
|  |  | PDCD6 has the prognostic significance in early stage ADC patients. | [107] |
| **PTGS2** | Prognostic | PTGS2 expression has insufficient evidence to be recommended as a prognostic marker in patients with colorectal cancer. | [108] |
|  |  | CD44 and PTGS2 methylation are independent prognostic markers for biochemical recurrence among prostate cancer patients. | [109] |
| **MDA** | Diagnostic | MDA and TBA-reactivity may be diagnostic indices of lipid peroxidation and peroxidative tissue injury. | [110] |
|  |  | Redox biomarkers can be potential diagnostic indicators of CRC advancement. | [111] |
|  | Prognostic | Blood and tissue NO and MDA are prognostic indicators of localized prostate cancer. | [112] |
|  |  | Serum MDA concentration was an independent risk factor for the stage of CRC, as well as an independent positive predictor of the depth of CR-wall invasion, carcinoma infiltration of lymph nodes, and the presence of metastasis. | [113] |
| **ACSL4** | Prognostic | The over-expression of ACSL4 will be a new potential therapeutic target for HCC as an independent adverse prognostic parameter. | [114] |
|  |  | High expression of ACSL4 predicted a worse prognosis in colorectal cancer, but predicted better prognosis in breast, brain and lung cancer. | [115] |
|  |  | The expression of ACSL4 may be a prognostic indicator and potential therapeutic target in BC. | [116] |
| **Beclin1** | Prognostic | Beclin1 and LC3 are associated with prognosis of MM. | [117] |
|  |  | Beclin1 expression of CTCs correlates with RCC prognosis. | [118] |
|  |  | High autophagic Beclin1 expression was an inferior prognostic marker for NPC. | [119] |
|  |  | Beclin1 and 4E-BP1 could be used as independent predictors of OS in ACRC patients treated with cetuximab. | [120] |
| **LDH** | Prognostic | LDH shows prognostic value in patients with metastatic prostate cancer. | [121] |
|  |  | LDH isoenzymes have profiles and prognostic values in patients with non-Hodgkin's lymphoma. | [122] |
|  |  | LDH has prognostic value in patients with hepatocellular carcinoma. | [123] |
|  |  | Serum LDH and serum ferritin are prognostic in children with neuroblastoma. | [124] |
|  |  | LDH serves as a prognostic marker in neoplastic meningitis. | [125] |
|  | Diagnostic and prognostic | The cancer/testis antigen LDH-C4 has diagnostic and prognostic value in breast cancer. | [126] |
| **RIPK** | Prognostic | RIPK4 may represent as an independent prognostic factor and a promising novel therapeutic target in keratinocyte differentiation and carcinogenesis. | [127] |
|  |  | RIP1K expression was shown to be higher in the malignant breast tumors than those of normal and benign breast tissues, which probably designates as a poor prognostic factor. | [128] |
| **LC3** | Diagnostic | MMP-2, LC3 and Beclin1 in EM guide the clinical diagnosis. | [129] |
|  | Diagnostic, Prognostic | CSF levels of LC3-B represented a potential diagnostic and prognostic biomarker of early-stage PD in patients. | [130] |
| **p62** | Prognostic | LC3-B and p62 could be used as prognostic biomarkers and potential therapeutic targets for gastric adenocarcinomas. | [131] |
|  |  | Low LC3-B and p62 expression show the most aggressive behavior and may be candidates for autophagy regulating therapeutics in esophageal adenocarcinomas. | [132] |
|  |  | p62 has an autophagy independent role with a pronounced prognostic impact of high p62 expression in pSQCC. | [133] |
|  |  | The expression of p-p62 and nuclear Nrf2 in ESCC influence the prognosis of ESCC. | [134] |
|  |  | The over-expression of p62 associated with poor OS can be used as a biomarker for lung cancer diagnosis and prognosis. | [135] |
| **HMGB** | Prognostic | p62, HMGB and LC3-B expression patterns may be linked to patient's prognosis in pSQCC. | [133] |
|  |  | Intracellular HMGB1 is a novel tumor suppressor with prognostic and therapeutic relevance in PDAC. | [136] |
|  |  | HMGB1, HMGB2, and HMGB3 may be useful prognostic indicators for patients with GC. | [137] |
|  |  | High ASMA (+) fibroblasts and low cytoplasmic HMGB1(+) breast cancer cells predict poor prognosis. | [138] |
|  |  | High expression of HMGB1 may become a prognostic and predictive marker for NSCLC. | [139] |
| **DNA fragmenta-tion** | Diagnostic | Active-caspase-3 and single-stranded DNA has become important diagnostic parameters in primary and metastatic liver tumors. | [140] |
|  | Prognostic | Circulating cell-free DNA could be used to monitor and improve the treatment of HCC. | [141] |
|  |  | DNA fragmentation factor (DFF45) expression is frequently upregulated in ovarian serous carcinomas and may serve as a marker of aggressive behavior with prognostic value. | [142] |
|  |  | The colocalization of DNA fragmentation and TTG have prognostic significance in enterovirus-associated DCM. | [143] |
|  |  | Cell-free DNA seems to be a useful prognostic marker in patients with BCA. | [144] |
| **ATG** | Prognostic | ATG-4B mRNA is an independent prognostic factor for hepatocellular carcinoma. | [145] |
|  |  | ATG proteins might serve as novel prognostic biomarkers in gastric cancer. | [146] |
|  |  | Expression of ATG-5 and MRP-1 may be independent prognostic markers for GC treatment. | [147] |
|  |  | ATG genes improve the clinical glioma management of LGG and assist individualized survival prediction. | [148] |
| **IL-18** | Prognostic | High expression of IL-18 is related with poor prognosis of human tumors and radiation-induced injuries. | [149] |
|  |  | Serum IL-18 level may be a useful marker to predict prognosis of patients with breast cancer in complete remission after surgery. | [150] |
|  |  | IL-18 is a biomarker for predicting prognosis in skin cutaneous melanoma. | [151] |
|  |  | IL-18 is an important non-invasive marker suspecting metastasis for breast carcinoma. | [152] |
|  | Diagnostic and prognostic | PSA, IL-18 and IL-10 prospectively have diagnostic and prognostic significance in patients with carcinoma prostate. | [153] |
| **NLRP3** | Diagnostic | The NLRP3 and related inflammasomes, and their regulated cytokines or receptors, may represent novel diagnostic or therapeutic targets in pulmonary diseases and other diseases. | [154] |
|  | Prognostic | NLRP3 expression was an independent prognostic factor for the survival of CRC patients. | [155] |
|  |  | High expression of NLRP3, NLRC4, and CASP1 in background non-tumorous liver is significantly correlated with poor prognosis of patients after resection of HCC. | [156] |
|  | Diagnostic and prognostic | NLRP3 could be a promising candidate diagnostic and prognostic target in human ESCC tissues. | [157] |
| **MLKL** | Prognostic | MLKL is a potential prognostic marker in gastric cancer. | [158] |
|  |  | MLKL expression may serve as a potential prognostic marker in patients with ovarian cancer. | [159] |
|  |  | MLKL is a prognostic biomarker for cervical squamous cell carcinoma. | [160] |
|  |  | MLKL expression in patients has the prognostic value of with PAC. | [161] |
|  |  | MLKL may provide important prognostic information in patients with colon cancer. | [162] |
| **miR-21** | Prognostic | miR-21 could be used as a prognostic biomarker in HNSCC patients. | [163] |
|  |  | Up-regulated miR-21 and down-regulated PDCD4 expression are associated with the aggressive progression and poor prognosis of stage II EC. | [164] |
|  | Diagnostic and prognostic | The expression of circulating miR-21 in plasma samples of oral cancer patients makes it a promising diagnostic and prognostic marker. | [165] |
|  |  | Circulating miR-130b- and miR-21 have diagnostic and therapeutic value for HCC patients. | [166] |
| **miR-137** | Prognostic | Downregulated serum miR-137 may be a potential non-invasive prognostic biomarker for poor prognosis in GBM patients. | [167] |
|  |  | High levels of miR-137 promoter methylation may have prognostic value for poor disease-free survival in NSCLC. | [168] |
|  |  | The expressions of miR-137 and AFM in combination significantly correlated with HCC progression suggests their potential as prognostic biomarkers for HCC. | [169] |
|  | Diagnostic and prognostic | Methylation silencing of miR-137 in colorectal adenomas has prognostic and therapeutic implications. | [170] |
| **miR-223** | Diagnostic | miR-223 is a novel potential diagnostic and therapeutic target for inflammatory disorders. | [171] |
|  | Diagnostic and prognostic | miR-223 is a potential diagnostic and prognostic marker for osteosarcoma. | [172] |
|  |  | Circulating miR-223-3p could represent a novel diagnostic and prognostic marker for HBV-related HCC. | [173] |
|  |  | Serum exosomal miR-223 is a potential diagnostic and prognostic biomarker for dementia. | [174] |
| **miR-184** | Diagnostic | The miR-184/SND1 axis may be a useful diagnostic and therapeutic tool for malignant glioma. | [175] |
|  | Prognostic | miR-184, miR-146b, miR-509-3 and LPAR5 are independent risk factors for prognosis of thyroid cancer. | [176] |
|  | Diagnostic and prognostic | miR-184 may provide a novel biomarker for the diagnosis, therapy, and prognosis of pancreatic ductal adenocarcinoma. | [177] |
|  |  | miR-184 acts as a potential diagnostic and prognostic marker in epithelial ovarian cancer. | [178] |

Abbreviations: GVHD: graft versus host disease, GTN: gestational trophoblastic neoplasia, NSCLC: non-small cell lung cancer, FL: follicular lymphoma, PIPO: pediatric intestinal pseudo-obstruction, DLBCL: diffuse large B-cell lymphoma, OSCC: oral squamous cell carcinoma, Bcl-6: B-cell lymphoma 6 protein, NLPHL: nodular lymphocyte-predominant Hodgkin lymphoma, CD10: cluster of differentiation 10, RLH: reactive lymphoid hyperplasia, cHL: classic Hodgkin lymphoma, HRS cells : Hodgkin and Reed-Sternberg cells, MDM2: murine double minute2, p53: tumor protein p53, CK20: Keratin 20, HGUC and LGUC: high-grade urothelial carcinoma and low-grade urothelial carcinoma, NF: neurofibroma, DM: desmoplastic melanoma, mLMS: myxoid leiomyosarcoma, IMT: inflammatory myofibroblastictumour, VSCC: vulvar squamous cell carcinoma, CRC: colorectal cancer, RB: retinoblastoma, SPTCL: subcutaneous panniculitis-like T-cell lymphoma, FDC: follicular dendritic cells, HL: Hodgkin lymphoma, CD95: cluster of differentiation 95, ALL: acute lymphoblastic leukemia, CC3: cleaved caspase-3, PPC: positive peritoneal cytology, ccRCC: clear cell renal cell carcinoma, BMSCC: buccal mucosa squamous cell carcinoma, PARP: poly (ADP-ribose) polymerase, BRCA: breast cancer, AML: acute myeloid leukemia, PTEN: phosphatase and tensin homolog, GST-pi: glutathione S-transferase Pi, RASSF1: Ras association domain-containing protein 1, PITX2: paired-like homeodomain transcription factor 2, EOC: epithelial ovarian carcinoma, NS: nephrotic syndrome, SCC: squamous cell carcinoma, HYNIC: hydrazinonicotinamide, ANAX2: Annexin A2(Annexin II), NASH: non-alcoholic steatohepatitis, BC: bladder cancer, M65: serum keratin 18, M30: serum ncaspase-cleaved cytokeratin, CK-18: cytokeratin 18, FGF-21: fibroblast growth factor 21, NAFLD: nonalcoholic fatty liver disease, ACLF: acute-on-chronic liver failure, HBV: hepatitis B virus, IAPs: inhibitor of apoptosis proteins, XIAP: X-linked inhibitor of apoptosis, EAC: esophageal adenocarcinoma, HNC: head and neck cancer, BIRC6: Baculoviral IAP Repeat Containing 6, IL-9: interleukin-9, TRAIL: tumor necrosis factor-related apoptosis-inducing ligand, TRAF-6: TNF receptor associated factor-6, PDAC: pancreatic ductal adenocarcinoma, PD-L1: programmed death ligand 1, c-FLIP: cellular FLICE (FADD-like IL-1β-converting enzyme)-inhibitory protein, HCC: hepatocellular carcinoma, PDCD6: programmed cell death 6 (PDCD6), ADC: adenocarcinoma, PTGS2/COX-2: prostaglandin endoperoxide synthase 2/Cyclooxygenase-2, CD44: cluster of differentiation-44, MDA: malondialdehyde, TBA: thiobarbituric acid, NO: nitric oxide, MM: multiple myeloma, CTCs: circulating tumor cells, NPC: nasopharyngeal carcinoma, 4E-BP1: 4E-binding protein 1, OS: overall survival, ACRC: advanced colorectal cancer, MMP-2: matrix metalloproteinase-2, EM: endometriosis, CSF: cerebrospinal fluid, PD: Parkinson's disease, BCA: bladder cancer, pSQCC: pulmonary squamous cell carcinoma, MRP-1: multidrug resistance-associated protein 1, HMGB: high-mobility group box, TTG: tissue-transglutaminase, DCM: dilated cardiomyopathy, PSA: prostate specific antigen, IL-10: interleukin-1, ESCC: esophageal squamous cell carcinoma, PAC: pancreatic adenocarcinoma, LGG: lower grade glioma, HNSCC: head and neck squamous cell cancer, PDCD4:programmed cell death 4, EC: esophageal carcinoma, GBM: glioblastoma, SND1: staphylococcal nuclease domain-containing 1, LPAR5: lysophosphatidic acid receptor 5.

1. Borges, L.R., et al., *Diagnosis of acute graft-versus-host disease in the gastrointestinal tract of patients undergoing allogeneic hematopoietic stem cell transplantation. A descriptive and critical study of diagnostic tests.* Hematol Transfus Cell Ther, 2020. **42**(3): p. 245-251.

2. Ross, W.A., *Treatment of Gastrointestinal Acute Graft-Versus-Host Disease.* Curr Treat Options Gastroenterol, 2005. **8**(3): p. 249-258.

3. Thompson, B., et al., *Prospective endoscopic evaluation for gastrointestinal graft-versus-host disease: determination of the best diagnostic approach.* Bone Marrow Transplant, 2006. **38**(5): p. 371-6.

4. Ross, W.A. and D. Couriel, *Colonic graft-versus-host disease.* Curr Opin Gastroenterol, 2005. **21**(1): p. 64-9.

5. Jia, Y., et al., *Apoptosis index correlates with chemotherapy efficacy and predicts the survival of patients with gastric cancer.* Tumour Biol, 2012. **33**(4): p. 1151-8.

6. Wu, A., et al., *Apoptosis and KI 67 index correlate with preoperative chemotherapy efficacy and better predict the survival of gastric cancer patients with combined therapy.* Cancer Chemother Pharmacol, 2014. **73**(5): p. 885-93.

7. Bhosle, S.M., N.G. Huilgol, and K.P. Mishra, *Apoptotic index as predictive marker for radiosensitivity of cervical carcinoma: evaluation of membrane fluidity, biochemical parameters and apoptosis after the first dose of fractionated radiotherapy to patients.* Cancer Detect Prev, 2005. **29**(4): p. 369-75.

8. Burcombe, R.J., et al., *Evaluation of ER, PgR, HER-2 and Ki-67 as predictors of response to neoadjuvant anthracycline chemotherapy for operable breast cancer.* Br J Cancer, 2005. **92**(1): p. 147-55.

9. Wang, X.P., et al., *Anti-tumor bioactivities of curcumin on mice loaded with gastric carcinoma.* Food Funct, 2017. **8**(9): p. 3319-3326.

10. Singh, A., et al., *Evaluation of expression of apoptosis-related proteins and their correlation with HPV, telomerase activity, and apoptotic index in cervical cancer.* Pathobiology, 2004. **71**(6): p. 314-22.

11. Khorsandi, L., et al., *Quercetin induces apoptosis and necroptosis in MCF-7 breast cancer cells.* Bratisl Lek Listy, 2017. **118**(2): p. 123-128.

12. Wu, X., et al., *Expressions of p53, c-MYC, BCL-2 and apoptotic index in human osteosarcoma and their correlations with prognosis of patients.* Cancer Epidemiol, 2012. **36**(2): p. 212-6.

13. Wu, X., et al., *Determination of the apoptotic index in osteosarcoma tissue and its relationship with patients prognosis.* Cancer Cell Int, 2013. **13**(1): p. 56.

14. Braga, A., et al., *Apoptotic index for prediction of postmolar gestational trophoblastic neoplasia.* Am J Obstet Gynecol, 2016. **215**(3): p. 336.e1-336.e12.

15. Negara, K.S., et al., *Protein 53 (P53) Expressions and Apoptotic Index of Amniotic Membrane Cells in the Premature Rupture of Membranes.* Open Access Maced J Med Sci, 2018. **6**(11): p. 1986-1992.

16. Dworakowska, D., et al., *Prognostic value of the apoptotic index analysed jointly with selected cell cycle regulators and proliferation markers in non-small cell lung cancer.* Lung Cancer, 2009. **66**(1): p. 127-33.

17. Gkogkou, C., et al., *Necrosis and apoptotic index as prognostic factors in non-small cell lung carcinoma: a review.* Springerplus, 2014. **3**: p. 120.

18. Weiss, L.M. and D. O'Malley, *Benign lymphadenopathies.* Mod Pathol, 2013. **26 Suppl 1**: p. S88-96.

19. Henrich, M., et al., *Lack of Bcl-2 expression in feline follicular lymphomas.* J Vet Diagn Invest, 2019. **31**(6): p. 809-817.

20. Weinberg, O.K., et al., *Low stage follicular lymphoma: biologic and clinical characterization according to nodal or extranodal primary origin.* Am J Surg Pathol, 2009. **33**(4): p. 591-8.

21. Ozsan, N., et al., *Clinicopathologic and genetic characterization of follicular lymphomas presenting in the ovary reveals 2 distinct subgroups.* Am J Surg Pathol, 2011. **35**(11): p. 1691-9.

22. Amiot, A., et al., *The role of immunohistochemistry in idiopathic chronic intestinal pseudoobstruction (CIPO): a case-control study.* Am J Surg Pathol, 2009. **33**(5): p. 749-58.

23. Park, S.H., et al., *Immunohistochemical studies of pediatric intestinal pseudo-obstruction: bcl2, a valuable biomarker to detect immature enteric ganglion cells.* Am J Surg Pathol, 2005. **29**(8): p. 1017-24.

24. Davids, M.S., et al., *Phase I First-in-Human Study of Venetoclax in Patients With Relapsed or Refractory Non-Hodgkin Lymphoma.* J Clin Oncol, 2017. **35**(8): p. 826-833.

25. Wang, X., et al., *Elucidating the Reaction Mechanisms between Triazine and Hydrogen Sulfide with pH Variation Using Mass Spectrometry.* Anal Chem, 2018. **90**(18): p. 11138-11145.

26. Pekarsky, Y., V. Balatti, and C.M. Croce, *BCL2 and miR-15/16: from gene discovery to treatment.* Cell Death Differ, 2018. **25**(1): p. 21-26.

27. Kelly, P.N. and A. Strasser, *The role of Bcl-2 and its pro-survival relatives in tumourigenesis and cancer therapy.* Cell Death Differ, 2011. **18**(9): p. 1414-24.

28. Huang, P., et al., *[Prognostic evaluation of P53 and BCL2 proteins in MYC/BCL2 double expression DLBCL].* Zhonghua Xue Ye Xue Za Zhi, 2019. **40**(7): p. 589-593.

29. Dwivedi, A., A. Mehta, and P. Solanki, *Evaluation of immunohistochemical subtypes in diffuse large B-cell lymphoma and its impact on survival.* Indian J Pathol Microbiol, 2015. **58**(4): p. 453-8.

30. Iqbal, J., et al., *BCL2 predicts survival in germinal center B-cell-like diffuse large B-cell lymphoma treated with CHOP-like therapy and rituximab.* Clin Cancer Res, 2011. **17**(24): p. 7785-95.

31. Kim, J.Y., et al., *Bcl-2 is a prognostic marker and its silencing inhibits recurrence in ameloblastomas.* Oral Dis, 2019. **25**(4): p. 1158-1168.

32. Katkoori, V.R., et al., *Bax expression is a candidate prognostic and predictive marker of colorectal cancer.* J Gastrointest Oncol, 2010. **1**(2): p. 76-89.

33. Zhang, M., et al., *Prognostic significance of Bcl-2 and Bax protein expression in the patients with oral squamous cell carcinoma.* J Oral Pathol Med, 2009. **38**(3): p. 307-13.

34. Wootipoom, V., et al., *Prognostic significance of Bax, Bcl-2, and p53 expressions in cervical squamous cell carcinoma treated by radiotherapy.* Gynecol Oncol, 2004. **94**(3): p. 636-42.

35. Pasqualucci, L., et al., *Molecular pathogenesis of non-Hodgkin's lymphoma: the role of Bcl-6.* Leuk Lymphoma, 2003. **44 Suppl 3**: p. S5-12.

36. Ohno, H., *Pathogenetic role of BCL6 translocation in B-cell non-Hodgkin's lymphoma.* Histol Histopathol, 2004. **19**(2): p. 637-50.

37. Chaganti, S.R., et al., *Involvement of BCL6 in chromosomal aberrations affecting band 3q27 in B-cell non-Hodgkin lymphoma.* Genes Chromosomes Cancer, 1998. **23**(4): p. 323-7.

38. de Leval, L., et al., *Cutaneous b-cell lymphomas of follicular and marginal zone types: use of Bcl-6, CD10, Bcl-2, and CD21 in differential diagnosis and classification.* Am J Surg Pathol, 2001. **25**(6): p. 732-41.

39. Dogan, A., et al., *CD10 and BCL-6 expression in paraffin sections of normal lymphoid tissue and B-cell lymphomas.* Am J Surg Pathol, 2000. **24**(6): p. 846-52.

40. Herbeck, R., et al., *B-cell transcription factors Pax-5, Oct-2, BOB.1, Bcl-6, and MUM1 are useful markers for the diagnosis of nodular lymphocyte predominant Hodgkin lymphoma.* Rom J Morphol Embryol, 2011. **52**(1): p. 69-74.

41. Kraus, M.D. and J. Haley, *Lymphocyte predominance Hodgkin's disease: the use of bcl-6 and CD57 in diagnosis and differential diagnosis.* Am J Surg Pathol, 2000. **24**(8): p. 1068-78.

42. Winter, J.N., et al., *Prognostic significance of Bcl-6 protein expression in DLBCL treated with CHOP or R-CHOP: a prospective correlative study.* Blood, 2006. **107**(11): p. 4207-13.

43. Chen, W.T., et al., *[Value of MYC, BCL-2 and BCL-6 for Evaluation of Prognosis in Patients with Diffuse Large B Cell Lymphoma].* Zhongguo Shi Yan Xue Ye Xue Za Zhi, 2019. **27**(2): p. 452-457.

44. Hans, C.P., et al., *Confirmation of the molecular classification of diffuse large B-cell lymphoma by immunohistochemistry using a tissue microarray.* Blood, 2004. **103**(1): p. 275-82.

45. Toon, C., et al., *Patterns of p53 immunoreactivity in non-neoplastic and neoplastic Barrett's mucosa of the oesophagus: in-depth evaluation in endoscopic mucosal resections.* Pathology, 2019. **51**(3): p. 253-260.

46. McKenney, J.K., et al., *Discriminatory immunohistochemical staining of urothelial carcinoma in situ and non-neoplastic urothelium: an analysis of cytokeratin 20, p53, and CD44 antigens.* Am J Surg Pathol, 2001. **25**(8): p. 1074-8.

47. Qin, J.J., et al., *Natural products targeting the p53-MDM2 pathway and mutant p53: Recent advances and implications in cancer medicine.* Genes Dis, 2018. **5**(3): p. 204-219.

48. Choi, S.Y., et al., *Diagnostic significance of dual immunocytochemical staining of p53/cytokeratin20 on liquid-based urine cytology to detect urothelial carcinoma.* Cytojournal, 2020. **17**: p. 3.

49. Elsensohn, A., et al., *Distinguishing Neurofibroma From Desmoplastic Melanoma: The Value of p53.* Am J Surg Pathol, 2018. **42**(3): p. 372-375.

50. Schaefer, I.M., et al., *Abnormal p53 and p16 staining patterns distinguish uterine leiomyosarcoma from inflammatory myofibroblastic tumour.* Histopathology, 2017. **70**(7): p. 1138-1146.

51. Nakamura, M., et al., *The Association and Significance of p53 in Gynecologic Cancers: The Potential of Targeted Therapy.* Int J Mol Sci, 2019. **20**(21).

52. Sand, F.L., et al., *The prognostic value of p16 and p53 expression for survival after vulvar cancer: A systematic review and meta-analysis.* Gynecol Oncol, 2019. **152**(1): p. 208-217.

53. Li, C., et al., *Prognostic value of p53 for colorectal cancer after surgical resection of pulmonary metastases.* World J Surg Oncol, 2016. **14**(1): p. 308.

54. Dai, X.L., et al., *Correlated expression of Fas, NF-kappaB, and VEGF-C in infiltrating ductal carcinoma of the breast.* Eur J Gynaecol Oncol, 2012. **33**(6): p. 633-9.

55. Blok, E.J., et al., *Combined evaluation of the FAS cell surface death receptor and CD8+ tumor infiltrating lymphocytes as a prognostic biomarker in breast cancer.* Oncotarget, 2017. **8**(9): p. 15610-15620.

56. Yamana, K., et al., *Prognostic impact of FAS/CD95/APO-1 in urothelial cancers: decreased expression of Fas is associated with disease progression.* Br J Cancer, 2005. **93**(5): p. 544-51.

57. Wang, W.S., et al., *Matrix metalloproteinase-7 increases resistance to Fas-mediated apoptosis and is a poor prognostic factor of patients with colorectal carcinoma.* Carcinogenesis, 2006. **27**(5): p. 1113-20.

58. Wu, G.Z., et al., *Clinicopathological significance of Fas and Fas ligand expressions in esophageal cancer.* Am J Cancer Res, 2015. **5**(9): p. 2865-71.

59. Shibakita, M., et al., *Prognostic significance of Fas and Fas ligand expressions in human esophageal cancer.* Clin Cancer Res, 1999. **5**(9): p. 2464-9.

60. Markovic, O., et al., *Clinical and prognostic significance of apoptotic profile in patients with newly diagnosed nodal diffuse large B-cell lymphoma (DLBCL).* Eur J Haematol, 2011. **86**(3): p. 246-55.

61. Takeshita, M., et al., *Clinicopathologic analysis of 22 cases of subcutaneous panniculitis-like CD56- or CD56+ lymphoma and review of 44 other reported cases.* Am J Clin Pathol, 2004. **121**(3): p. 408-16.

62. Verbeke, C.S., et al., *Fas ligand expression in Hodgkin lymphoma.* Am J Surg Pathol, 2001. **25**(3): p. 388-94.

63. Heng, B., et al., *Diagnostic Performance of Fas Ligand mRNA Expression for Acute Rejection after Kidney Transplantation: A Systematic Review and Meta-Analysis.* PLoS One, 2016. **11**(11): p. e0165628.

64. Montiel-Cervantes, L.A., et al., *Prognostic Value of CD95, Active Caspase-3, and Bcl-2 Expression in Adult Patients with De Novo Acute Lymphoblastic Leukemia.* Arch Med Res, 2018. **49**(1): p. 44-50.

65. Huang, K.H., et al., *Caspase-3, a key apoptotic protein, as a prognostic marker in gastric cancer after curative surgery.* Int J Surg, 2018. **52**: p. 258-263.

66. Singh, P., et al., *Interleukin-1beta and Caspase-3 expression serve as independent prognostic markers for metastasis and survival in oral squamous cell carcinoma.* Cancer Biomark, 2019. **26**(1): p. 109-122.

67. Ogane, N., et al., *Cleaved caspase-3 expression is a potential prognostic factor for endometrial cancer with positive peritoneal cytology.* Cytopathology, 2018. **29**(3): p. 254-261.

68. Vilella-Arias, S.A., et al., *Loss of caspase 7 expression is associated with poor prognosis in renal cell carcinoma clear cell subtype.* Urology, 2013. **82**(4): p. 974.e1-7.

69. Yao, Q., et al., *Synergistic role of Caspase-8 and Caspase-3 expressions: Prognostic and predictive biomarkers in colorectal cancer.* Cancer Biomark, 2018. **21**(4): p. 899-908.

70. Sträter, J., et al., *Expression and prognostic significance of APAF-1, caspase-8 and caspase-9 in stage II/III colon carcinoma: caspase-8 and caspase-9 is associated with poor prognosis.* Int J Cancer, 2010. **127**(4): p. 873-80.

71. Kuhlmann, J.D., et al., *Prognostic relevance of caspase 8 -652 6N InsDel and Asp302His polymorphisms for breast cancer.* BMC Cancer, 2016. **16**: p. 618.

72. Bräutigam, K., et al., *PARP-1 expression as a prognostic factor in Desmoid-type fibromatosis.* Ann Diagn Pathol, 2020. **44**: p. 151442.

73. Gan, A., et al., *Poly(adenosine diphosphate-ribose) polymerase expression in BRCA-proficient ovarian high-grade serous carcinoma; association with patient survival.* Hum Pathol, 2013. **44**(8): p. 1638-47.

74. Pashaiefar, H., et al., *PARP-1 Overexpression as an Independent Prognostic Factor in Adult Non-M3 Acute Myeloid Leukemia.* Genet Test Mol Biomarkers, 2018. **22**(6): p. 343-349.

75. Siraj, A.K., et al., *Overexpression of PARP is an independent prognostic marker for poor survival in Middle Eastern breast cancer and its inhibition can be enhanced with embelin co-treatment.* Oncotarget, 2018. **9**(99): p. 37319-37332.

76. Egevad, L., et al., *Contemporary prognostic indicators for prostate cancer incorporating International Society of Urological Pathology recommendations.* Pathology, 2018. **50**(1): p. 60-73.

77. Hjortkjær, M., et al., *The Prognostic Value of BRCA1 and PARP Expression in Epithelial Ovarian Carcinoma: Immunohistochemical Detection.* Int J Gynecol Pathol, 2017. **36**(2): p. 180-189.

78. Simsek, B., et al., *Urinary annexin V in children with nephrotic syndrome: a new prognostic marker?* Pediatr Nephrol, 2008. **23**(1): p. 79-82.

79. Loose, D., et al., *Prognostic value of 99mTc-HYNIC annexin-V imaging in squamous cell carcinoma of the head and neck.* Eur J Nucl Med Mol Imaging, 2008. **35**(1): p. 47-52.

80. Zhuang, C., et al., *Expression levels and prognostic values of annexins in liver cancer.* Oncol Lett, 2019. **18**(6): p. 6657-6669.

81. Xu, B., et al., *Gasdermin D plays a key role as a pyroptosis executor of non-alcoholic steatohepatitis in humans and mice.* J Hepatol, 2018. **68**(4): p. 773-782.

82. Gao, J., et al., *Downregulation of GSDMD attenuates tumor proliferation via the intrinsic mitochondrial apoptotic pathway and inhibition of EGFR/Akt signaling and predicts a good prognosis in non‑small cell lung cancer.* Oncol Rep, 2018. **40**(4): p. 1971-1984.

83. Peng, J., et al., *CD147 Expression Is Associated with Tumor Proliferation in Bladder Cancer via GSDMD.* Biomed Res Int, 2020. **2020**: p. 7638975.

84. Sen, F., et al., *Diagnostic value of serum M30 and M65 in patients with nasopharyngeal carcinoma.* Tumour Biol, 2015. **36**(2): p. 1039-44.

85. He, L., et al., *Diagnostic Value of CK-18, FGF-21, and Related Biomarker Panel in Nonalcoholic Fatty Liver Disease: A Systematic Review and Meta-Analysis.* Biomed Res Int, 2017. **2017**: p. 9729107.

86. Zheng, S.J., et al., *Prognostic value of M30/M65 for outcome of hepatitis B virus-related acute-on-chronic liver failure.* World J Gastroenterol, 2014. **20**(9): p. 2403-11.

87. Yaman, E., et al., *Serum M30 levels are associated with survival in advanced gastric carcinoma patients.* Int Immunopharmacol, 2010. **10**(7): p. 719-22.

88. Schiffmann, L.M., et al., *Elevated X-linked inhibitor of apoptosis protein (XIAP) expression uncovers detrimental prognosis in subgroups of neoadjuvant treated and T-cell rich esophageal adenocarcinoma.* Bmc Cancer, 2019. **19**.

89. Farnebo, L., et al., *Combining factors on protein and gene level to predict radioresponse in head and neck cancer cell lines.* J Oral Pathol Med, 2011. **40**(10): p. 739-46.

90. Veiga, G.L.D., et al., *The role of Survivin as a biomarker and potential prognostic factor for breast cancer.* Rev Assoc Med Bras (1992), 2019. **65**(6): p. 893-901.

91. Zhuang, W., et al., *Baculoviral IAP Repeat Containing 6 (BIRC6) Is a Predictor of Prognosis in Prostate Cancer.* Med Sci Monit, 2018. **24**: p. 839-845.

92. Chen, X., et al., *Expression of the IAP protein family acts cooperatively to predict prognosis in human bladder cancer patients.* Oncol Lett, 2013. **5**(4): p. 1278-1284.

93. Moledina, D.G., et al., *Urine TNF-α and IL-9 for clinical diagnosis of acute interstitial nephritis.* JCI Insight, 2019. **4**(10).

94. Nakayama, S., et al., *TNF-α receptor 1 expression predicts poor prognosis of diffuse large B-cell lymphoma, not otherwise specified.* Am J Surg Pathol, 2014. **38**(8): p. 1138-46.

95. Heilmann, T., et al., *Heterogeneous intracellular TRAIL-receptor distribution predicts poor outcome in breast cancer patients.* J Mol Med (Berl), 2019. **97**(8): p. 1155-1167.

96. Gundlach, J.P., et al., *Cytoplasmic TRAIL-R1 is a positive prognostic marker in PDAC.* BMC Cancer, 2018. **18**(1): p. 777.

97. Han, F., et al., *TRAF6 promotes the invasion and metastasis and predicts a poor prognosis in gastric cancer.* Pathol Res Pract, 2016. **212**(1): p. 31-7.

98. Bilir, C., et al., *Increased serum tumor necrosis factor receptor-associated factor-6 expression in patients with non-metastatic triple-negative breast cancer.* Oncol Lett, 2015. **9**(6): p. 2819-2824.

99. Zhang, M., et al., *Expression of PD-L1 and prognosis in breast cancer: a meta-analysis.* Oncotarget, 2017. **8**(19): p. 31347-31354.

100. Qiu, Y.J. and M.Z. Zhang, *[PD-L1 expression is related with prognosis of small cell lung cancer].* Zhonghua Zhong Liu Za Zhi, 2019. **41**(3): p. 214-217.

101. Zang, F., X. Wei, and B. Sun, *[Relationship of c-FLIP(L) protein expression with molecular subtyping and clinical prognosis in invasive breast cancer].* Zhonghua Bing Li Xue Za Zhi, 2014. **43**(7): p. 442-6.

102. Korkolopoulou, P., et al., *c-FLIP expression in colorectal carcinomas: association with Fas/FasL expression and prognostic implications.* Histopathology, 2007. **51**(2): p. 150-6.

103. Guerriero, E., et al., *GPX4 and GPX7 over-expression in human hepatocellular carcinoma tissues.* Eur J Histochem, 2015. **59**(4): p. 2540.

104. Kinowaki, Y., et al., *Glutathione peroxidase 4 overexpression inhibits ROS-induced cell death in diffuse large B-cell lymphoma.* Lab Invest, 2018. **98**(5): p. 609-619.

105. Zhao, H., et al., *Gpx 4 is involved in the proliferation, migration and apoptosis of glioma cells.* Pathol Res Pract, 2017. **213**(6): p. 626-633.

106. Yoon, J.H., et al., *Programmed cell death 6 (PDCD6) as a prognostic marker for gastric cancers.* Tumour Biol, 2012. **33**(2): p. 485-94.

107. Aviel-Ronen, S., et al., *Genomic markers for malignant progression in pulmonary adenocarcinoma with bronchioloalveolar features.* Proc Natl Acad Sci U S A, 2008. **105**(29): p. 10155-60.

108. Kunzmann, A.T., et al., *PTGS2 (Cyclooxygenase-2) expression and survival among colorectal cancer patients: a systematic review.* Cancer Epidemiol Biomarkers Prev, 2013. **22**(9): p. 1490-7.

109. Woodson, K., et al., *CD44 and PTGS2 methylation are independent prognostic markers for biochemical recurrence among prostate cancer patients with clinically localized disease.* Epigenetics, 2006. **1**(4): p. 183-6.

110. Janero, D.R., *Malondialdehyde and thiobarbituric acid-reactivity as diagnostic indices of lipid peroxidation and peroxidative tissue injury.* Free Radic Biol Med, 1990. **9**(6): p. 515-40.

111. Zińczuk, J., et al., *Antioxidant Barrier, Redox Status, and Oxidative Damage to Biomolecules in Patients with Colorectal Cancer. Can Malondialdehyde and Catalase Be Markers of Colorectal Cancer Advancement?* Biomolecules, 2019. **9**(10).

112. Dillioglugil, M.O., et al., *Blood and tissue nitric oxide and malondialdehyde are prognostic indicators of localized prostate cancer.* Int Urol Nephrol, 2012. **44**(6): p. 1691-6.

113. Rašić, I., et al., *THE RELATIONSHIP BETWEEN SERUM LEVEL OF MALONDIALDEHYDE AND PROGRESSION OF COLORECTAL CANCER.* Acta Clin Croat, 2018. **57**(3): p. 411-416.

114. Sun, X.J. and G.L. Xu, *Overexpression of Acyl-CoA Ligase 4 (ACSL4) in Patients with Hepatocellular Carcinoma and its Prognosis.* Med Sci Monit, 2017. **23**: p. 4343-4350.

115. Chen, W.C., et al., *Systematic Analysis of Gene Expression Alterations and Clinical Outcomes for Long-Chain Acyl-Coenzyme A Synthetase Family in Cancer.* PLoS One, 2016. **11**(5): p. e0155660.

116. Dinarvand, N., et al., *Evaluation of long-chain acyl-coenzyme A synthetase 4 (ACSL4) expression in human breast cancer.* Res Pharm Sci, 2020. **15**(1): p. 48-56.

117. Jung, G., et al., *Autophagic Markers BECLIN 1 and LC3 are Associated with Prognosis of Multiple Myeloma.* Acta Haematol, 2015. **134**(1): p. 17-24.

118. Wang, Z.L., et al., *Dynamic changes of different phenotypic and genetic circulating tumor cells as a biomarker for evaluating the prognosis of RCC.* Cancer Biol Ther, 2019. **20**(4): p. 505-512.

119. Wan, X.B., et al., *Elevated Beclin 1 expression is correlated with HIF-1alpha in predicting poor prognosis of nasopharyngeal carcinoma.* Autophagy, 2010. **6**(3): p. 395-404.

120. Guo, G.F., et al., *Predictive and prognostic implications of 4E-BP1, Beclin-1, and LC3 for cetuximab treatment combined with chemotherapy in advanced colorectal cancer with wild-type KRAS: Analysis from real-world data.* World J Gastroenterol, 2019. **25**(15): p. 1840-1853.

121. Mori, K., et al., *Prognostic Value of Lactate Dehydrogenase in Metastatic Prostate Cancer: A Systematic Review and Meta-analysis.* Clin Genitourin Cancer, 2019. **17**(6): p. 409-418.

122. Dumontet, C., et al., *Profiles and prognostic values of LDH isoenzymes in patients with non-Hodgkin's lymphoma.* Leukemia, 1999. **13**(5): p. 811-7.

123. Kong, W., et al., *Prognostic Value of Lactate Dehydrogenase in Patients with Hepatocellular Carcinoma: A Meta-Analysis.* Biomed Res Int, 2018. **2018**: p. 1723184.

124. Moroz, V., et al., *The prognostic strength of serum LDH and serum ferritin in children with neuroblastoma: A report from the International Neuroblastoma Risk Group (INRG) project.* Pediatr Blood Cancer, 2020. **67**(8): p. e28359.

125. Cacho-Díaz, B., et al., *Lactate dehydrogenase as a prognostic marker in neoplastic meningitis.* J Clin Neurosci, 2018. **51**: p. 39-42.

126. Cui, Z., et al., *Diagnostic and prognostic value of the cancer-testis antigen lactate dehydrogenase C4 in breast cancer.* Clin Chim Acta, 2020. **503**: p. 203-209.

127. Xu, J., Q. Wei, and Z. He, *Insight Into the Function of RIPK4 in Keratinocyte Differentiation and Carcinogenesis.* Front Oncol, 2020. **10**: p. 1562.

128. Karami-Tehrani, F., et al., *Evaluation of RIP1K and RIP3K expressions in the malignant and benign breast tumors.* Tumour Biol, 2016. **37**(7): p. 8849-56.

129. Sui, X., et al., *Expression and significance of autophagy genes LC3, Beclin1 and MMP-2 in endometriosis.* Exp Ther Med, 2018. **16**(3): p. 1958-1962.

130. Youn, J., et al., *Cerebrospinal Fluid Levels of Autophagy-related Proteins Represent Potentially Novel Biomarkers of Early-Stage Parkinson's Disease.* Sci Rep, 2018. **8**(1): p. 16866.

131. Kim, J.S., et al., *Prognostic Significance of LC3B and p62/SQSTM1 Expression in Gastric Adenocarcinoma.* Anticancer Res, 2019. **39**(12): p. 6711-6722.

132. Adams, O., et al., *Prognostic relevance of autophagy markers LC3B and p62 in esophageal adenocarcinomas.* Oncotarget, 2016. **7**(26): p. 39241-39255.

133. Langer, R., et al., *Expression Analysis of Autophagy Related Markers LC3B, p62 and HMGB1 Indicate an Autophagy-Independent Negative Prognostic Impact of High p62 Expression in Pulmonary Squamous Cell Carcinomas.* Cancers (Basel), 2018. **10**(9).

134. Wang, Z., et al., *The expression of p-p62 and nuclear Nrf2 in esophageal squamous cell carcinoma and association with radioresistance.* Thorac Cancer, 2020. **11**(1): p. 130-139.

135. Wang, B.J., et al., *Expression of autophagy-related factor p62 for lung cancer diagnosis and prognosis: A systematic review and meta-analysis.* Math Biosci Eng, 2019. **16**(6): p. 6805-6821.

136. Kang, R., et al., *Intracellular HMGB1 as a novel tumor suppressor of pancreatic cancer.* Cell Res, 2017. **27**(7): p. 916-932.

137. Fang, J., et al., *Bioinformatics analysis of the prognosis and biological significance of HMGB1, HMGB2, and HMGB3 in gastric cancer.* J Cell Physiol, 2020. **235**(4): p. 3438-3446.

138. Amornsupak, K., et al., *High ASMA(+) Fibroblasts and Low Cytoplasmic HMGB1(+) Breast Cancer Cells Predict Poor Prognosis.* Clin Breast Cancer, 2017. **17**(6): p. 441-452.e2.

139. Wang, J.L., et al., *Expression of high mobility group box - B1 (HMGB-1) and matrix metalloproteinase-9 (MMP-9) in non-small cell lung cancer (NSCLC).* Asian Pac J Cancer Prev, 2014. **15**(12): p. 4865-9.

140. Karamitopoulou, E., et al., *Active caspase 3 and DNA fragmentation as markers for apoptotic cell death in primary and metastatic liver tumours.* Pathology, 2007. **39**(6): p. 558-64.

141. Mezzalira, S., et al., *Circulating-Free DNA Analysis in Hepatocellular Carcinoma: A Promising Strategy to Improve Patients' Management and Therapy Outcomes.* Int J Mol Sci, 2019. **20**(21).

142. Brustmann, H., *DNA fragmentation factor (DFF45): expression and prognostic value in serous ovarian cancer.* Pathol Res Pract, 2006. **202**(10): p. 713-20.

143. Lotze, U., et al., *Damaged myocytes as detected by the colocalization of DNA fragmentation and tissue transglutaminase and their prognostic significance in enterovirus-associated dilated cardiomyopathy.* Eur J Clin Invest, 2001. **31**(9): p. 744-55.

144. Ellinger, J., et al., *Apoptotic DNA fragments in serum of patients with muscle invasive bladder cancer: a prognostic entity.* Cancer Lett, 2008. **264**(2): p. 274-80.

145. Ali, M.A., et al., *Investigating miRNA-661 and ATG4-B mRNA expression as potential biomarkers for hepatocellular carcinoma.* Biomark Med, 2018. **12**(3): p. 245-256.

146. Cao, Q.H., et al., *Prognostic value of autophagy related proteins ULK1, Beclin 1, ATG3, ATG5, ATG7, ATG9, ATG10, ATG12, LC3B and p62/SQSTM1 in gastric cancer.* Am J Transl Res, 2016. **8**(9): p. 3831-3847.

147. Ge, J., et al., *Upregulation of autophagy-related gene-5 (ATG-5) is associated with chemoresistance in human gastric cancer.* PLoS One, 2014. **9**(10): p. e110293.

148. Wang, C., et al., *Prognostic model and nomogram construction based on autophagy signatures in lower grade glioma.* J Cell Physiol, 2020.

149. Yao, Z., et al., *Prognostic Role of IL-18 in Various Human Cancers and Radiation Injuries: A Meta-Analysis.* Dose Response, 2020. **18**(2): p. 1559325820931360.

150. Günel, N., et al., *Prognostic value of serum IL-18 and nitric oxide activity in breast cancer patients at operable stage.* Am J Clin Oncol, 2003. **26**(4): p. 416-21.

151. Gil, M. and K.E. Kim, *Interleukin-18 Is a Prognostic Biomarker Correlated with CD8(+) T Cell and Natural Killer Cell Infiltration in Skin Cutaneous Melanoma.* J Clin Med, 2019. **8**(11).

152. Eissa, S.A., et al., *Importance of serum IL-18 and RANTES as markers for breast carcinoma progression.* J Egypt Natl Canc Inst, 2005. **17**(1): p. 51-5.

153. Dwivedi, S., et al., *Diagnostic and prognostic significance of prostate specific antigen and serum interleukin 18 and 10 in patients with locally advanced prostate cancer: a prospective study.* Asian Pac J Cancer Prev, 2011. **12**(7): p. 1843-8.

154. Lee, S., et al., *Regulation and Function of the Nucleotide Binding Domain Leucine-Rich Repeat-Containing Receptor, Pyrin Domain-Containing-3 Inflammasome in Lung Disease.* Am J Respir Cell Mol Biol, 2016. **54**(2): p. 151-60.

155. Wang, B., et al., *The association of aberrant expression of NLRP3 and p-S6K1 in colorectal cancer.* Pathol Res Pract, 2020. **216**(1): p. 152737.

156. Sonohara, F., et al., *Association of Inflammasome Components in Background Liver with Poor Prognosis After Curatively-resected Hepatocellular Carcinoma.* Anticancer Res, 2017. **37**(1): p. 293-300.

157. Yu, S., et al., *Activation of NLRP3 inflammasome promotes the proliferation and migration of esophageal squamous cell carcinoma.* Oncol Rep, 2020. **43**(4): p. 1113-1124.

158. Sun, W., et al., *MLKL is a potential prognostic marker in gastric cancer.* Oncol Lett, 2019. **18**(4): p. 3830-3836.

159. He, L., et al., *Low expression of mixed lineage kinase domain-like protein is associated with poor prognosis in ovarian cancer patients.* Onco Targets Ther, 2013. **6**: p. 1539-43.

160. Ruan, J., et al., *Mixed lineage kinase domain-like protein is a prognostic biomarker for cervical squamous cell cancer.* Int J Clin Exp Pathol, 2015. **8**(11): p. 15035-8.

161. Colbert, L.E., et al., *Pronecrotic mixed lineage kinase domain-like protein expression is a prognostic biomarker in patients with early-stage resected pancreatic adenocarcinoma.* Cancer, 2013. **119**(17): p. 3148-55.

162. Li, X., et al., *Association of Mixed Lineage Kinase Domain-Like Protein Expression With Prognosis in Patients With Colon Cancer.* Technol Cancer Res Treat, 2017. **16**(4): p. 428-434.

163. Irimie-Aghiorghiesei, A.I., et al., *Prognostic Value of MiR-21: An Updated Meta-Analysis in Head and Neck Squamous Cell Carcinoma (HNSCC).* J Clin Med, 2019. **8**(12).

164. Zhang, J., et al., *Prognostic significance of miR-21 and PDCD4 in patients with stage II esophageal carcinoma after surgical resection.* J Cell Biochem, 2018. **119**(6): p. 4783-4791.

165. Mahmood, N., et al., *Circulating miR-21 as a prognostic and predictive biomarker in oral squamous cell carcinoma.* Pak J Med Sci, 2019. **35**(5): p. 1408-1412.

166. Zhang, N., et al., *Circulating miR-130b- and miR-21-based diagnostic markers and therapeutic targets for hepatocellular carcinoma.* Mol Genet Genomic Med, 2019. **7**(12): p. e1012.

167. Li, H.Y., et al., *Circulating microRNA-137 is a potential biomarker for human glioblastoma.* Eur Rev Med Pharmacol Sci, 2016. **20**(17): p. 3599-604.

168. Min, L., et al., *Aberrant microRNA-137 promoter methylation is associated with lymph node metastasis and poor clinical outcomes in non-small cell lung cancer.* Oncol Lett, 2018. **15**(5): p. 7744-7750.

169. Wei, Q., et al., *Prognostic relevance of miR-137 and its liver microenvironment regulatory target gene AFM in hepatocellular carcinoma.* J Cell Physiol, 2019. **234**(7): p. 11888-11899.

170. Balaguer, F., et al., *Epigenetic silencing of miR-137 is an early event in colorectal carcinogenesis.* Cancer Res, 2010. **70**(16): p. 6609-18.

171. Aziz, F., *The emerging role of miR-223 as novel potential diagnostic and therapeutic target for inflammatory disorders.* Cell Immunol, 2016. **303**: p. 1-6.

172. Dong, J., et al., *miRNA-223 is a potential diagnostic and prognostic marker for osteosarcoma.* J Bone Oncol, 2016. **5**(2): p. 74-9.

173. Pratedrat, P., et al., *Diagnostic and prognostic roles of circulating miRNA-223-3p in hepatitis B virus-related hepatocellular carcinoma.* PLoS One, 2020. **15**(4): p. e0232211.

174. Wei, H., et al., *Serum Exosomal miR-223 Serves as a Potential Diagnostic and Prognostic Biomarker for Dementia.* Neuroscience, 2018. **379**: p. 167-176.

175. Emdad, L., et al., *Suppression of miR-184 in malignant gliomas upregulates SND1 and promotes tumor aggressiveness.* Neuro Oncol, 2015. **17**(3): p. 419-29.

176. Tang, J., et al., *Bioinformatic analysis and identification of potential prognostic microRNAs and mRNAs in thyroid cancer.* PeerJ, 2018. **6**: p. e4674.

177. Li, S., et al., *Effect of miR-184 on Proliferation and Apoptosis of Pancreatic Ductal Adenocarcinoma and Its Mechanism.* Technol Cancer Res Treat, 2020. **19**: p. 1533033820943237.

178. Qin, C.Z., et al., *MicroRNA-184 acts as a potential diagnostic and prognostic marker in epithelial ovarian cancer and regulates cell proliferation, apoptosis and inflammation.* Pharmazie, 2015. **70**(10): p. 668-73.
